# Genome-Wide Analysis of the Complex Transcriptional Networks of Rice Developing Seeds

**DOI:** 10.1371/journal.pone.0031081

**Published:** 2012-02-17

**Authors:** Liang-Jiao Xue, Jing-Jing Zhang, Hong-Wei Xue

**Affiliations:** National Key Laboratory of Plant Molecular Genetics, Institute of Plant Physiology and Ecology, Shanghai Institutes for Biological Sciences, Chinese Academy of Sciences, Shanghai, China; Instituto de Biología Molecular y Celular de Plantas, Spain

## Abstract

**Background:**

The development of rice (*Oryza sativa*) seed is closely associated with assimilates storage and plant yield, and is fine controlled by complex regulatory networks. Exhaustive transcriptome analysis of developing rice embryo and endosperm will help to characterize the genes possibly involved in the regulation of seed development and provide clues of yield and quality improvement.

**Principal Findings:**

Our analysis showed that genes involved in metabolism regulation, hormone response and cellular organization processes are predominantly expressed during rice development. Interestingly, 191 transcription factor (TF)-encoding genes are predominantly expressed in seed and 59 TFs are regulated during seed development, some of which are homologs of seed-specific TFs or regulators of *Arabidopsis* seed development. Gene co-expression network analysis showed these TFs associated with multiple cellular and metabolism pathways, indicating a complex regulation of rice seed development. Further, by employing a cold-resistant *cultivar* Hanfeng (HF), genome-wide analyses of seed transcriptome at normal and low temperature reveal that rice seed is sensitive to low temperature at early stage and many genes associated with seed development are down-regulated by low temperature, indicating that the delayed development of rice seed by low temperature is mainly caused by the inhibition of the development-related genes. The transcriptional response of seed and seedling to low temperature is different, and the differential expressions of genes in signaling and metabolism pathways may contribute to the chilling tolerance of HF during seed development.

**Conclusions:**

These results provide informative clues and will significantly improve the understanding of rice seed development regulation and the mechanism of cold response in rice seed.

## Introduction

Rice seed (strictly caryopsis) is a highly specialized storage organ for nutrient materials including carbohydrates, proteins, and lipids. Study of the regulatory networks of seed development is of significance for understanding the mechanisms controlling plant reproductive development and crop improvement.

The grass caryopsis is a single-seeded fruit composed of three genetically distinct tissue types: the filial embryo, the triploid endosperm, and the maternal pericarp and seed coat. A staging system of rice embryo development, containing 10 stages, had been proposed based on the landmark events during embryo development [1]. Embryonic shoot apical meristem (SAM) is formed during early embryogenesis and is the center of organogenesis during post-embryonic development [2]. SAM differentiates after late globular stage (3 days after fertilization, DAF), and first leaf primordium emerges at 5–6 DAF. After formation of the second and third leaf primordial at 7–8 DAF, seed organs enlarge and morphogenesis completes at 9–10 DAF, and embryo matures at 11–20 DAF [1]. Genetic and molecular mechanisms governing embryonic SAM formation have been extensively investigated and several key genetic components have been characterized, including OSH1, a class I Knotted1-like homeobox (KNOX) transcription factor that involves in embryonic SAM formation and maintenance [2].

The development of rice endosperm belongs to the *ab initio* nuclear type. At early days after fertilization, a large number of free endosperm nuclei locate in the peripheral region of embryo sac. Cell wall formation occurs centripetally at 3 DAF and then mitotic phase starts at 4 DAF [3]. Cellularization of endosperm is completed at approximately 6 DAF followed by endoreduplication at 8–10 DAF [3]. Programmed cell death (PCD) in the endosperm begins at 16 DAF, and as a result, only the cells in aleurone layer are alive in the mature endosperm [3].

Many efforts have been made to characterize the regulatory mechanism of seed development. A large number of mutants with alteration in various aspects of rice seeds have been reported (reviewed by Kurata et al., [4]) and many mutants with defective embryo development are caused by the mutation of genes regulating SAM formation. Mutations of rice *SHOOTLESS2* (*SHL2*), *SHL4*/*SHOOT ORGANIZATION2* (*SHO2*), and *SHO1* result in the defective expression of homeodomain-leucine zipper (HD-ZIPIII) family transcription factors (TFs), which play important roles in SAM initiation and maintenance during embryo development [5]. In addition, genes involved in starch and storage protein synthesis and accumulation are identified by studying the endosperm mutants, including *Waxy* (*Wx*) and *Gulutelin1* (encoding glutelin precursors) genes [4].

Plant hormones are key regulators of seed development. Deficiency of indole-3-acetic acid (IAA) results in the lethal embryo during embryogenesis [6]. Genes encoding late embryogenesis abundant (LEA) proteins are activated by abscisic acid through OsVP1 to protect embryo in dehydration process [7]. Cytokinin, gibberellic acid and brassinosteroids are also involved in the regulation of seed development [8].

Systemic transcriptomic analyses are of great help to illustrate the transcriptional network of seed development, which has been performed in *Arabidopsis* [9], maize [10], [11], wheat [12], barley [13] and rice [14]. However, most of the studies focused on various metabolic pathways such as nutrient partitioning. In rice, different technologies including EST [15], [16], cDNA [16] and DNA microarray [14], [17], [18], and deep sequencing [19] have been applied to analyze the transcriptional profiles in seeds. In most of these studies (except in 14), RNAs were collected from seed tissues at single time point of multiple genotypes/cultivars. For example, genes required for grain endosperm chalkiness determination are identified by comparing the seed transcriptomes of high chalkiness near-isogenic line and its normal parental line [17] and genes related to grain quality are identified by comparing five rice cultivars through deep sequencing [19]. In addition, characterization of the transcriptional dynamics during seed development provides informative clues of gene regulation and a *cis* element, AACA, is identified to be over-represented in genes up-regulated during grain filling (MYB TFs are possibly involved in the regulation of seed development through similar cis-elements [14]). Our previous studies by using cDNA microarray indicated that homeobox TFs are actively expressed in developing rice seed, suggesting the essential roles of them in regulation of seed development [20].

As a species mostly planted in tropical and subtropical regions, rice is sensitive to chilling temperature (0–15°C), especially at reproductive stage, which is a serious problem at high latitudes and in uplands at low latitudes. During bolting and flowering stages, low temperature causes a significant decline in spikelet fertility due to the failure of microspore development [21]. However, the effect of low temperature on seed development has not been well characterized, although several TFs have been identified being crucial in cold response during vegetative stage [22]. C-repeat (CRT)-binding factors (CBFs)/dehydration-responsive-element-binding proteins (DREBs), which belong to APETALA2 (AP2)/Ethylene Response Factor (ERF) family, are induced during cold acclimation and activate the transcription of cold-responsive genes, including COR (cold regulated), KIN (cold-induced), LTI (low-temperature induced) and RD (responsive to dehydration) genes, through binding to their promoters [22]. Some TFs, such as Inducer of CBF Expression1 (ICE1) and AtMYB15, regulate the expression of CBF/DREB genes [23]. However, there are still few studies on the transcriptional regulation by low temperature in rice seeds.

To characterize the transcriptional networks controlling rice seed development, especially at low temperature, we analyzed the transcriptome of developing embryo and endosperm, and seeds under chilling temperature using Affymetrix rice genome array. Our results showed that genes associated with transcriptional regulation, signaling pathways and metabolic pathways were involved in seed development and the response to low temperature at early stages of seed development, which will provide informative clues on the transcriptional control of rice seed development.

## Results

### Preferentially expressed genes and biological processes associated with rice seed development

To characterize the transcriptional dynamics during seed development, RNAs from Zhonghua 11 (ZH, japonica cultivar) seeds at four developmental stages of embryo and endosperm respectively were extracted, including embryo at 3, 6, 9 and 12 days after fertilization (DAF) and endosperm at 3, 6, 9 and 16 DAF. These developmental stages correspond to the important landmark events during embryo [1] and endosperm development [3]. Rice tissues of leaf, root, seedling, and ovary were selected to compare with the transcription profile of embryo and endosperm. These twelve samples were collected and two biological replicates were performed for each sample. Calculations of the Pearson correlation coefficients (PCCs) of each sample after hybridization and normalization revealed the high replication quality of hybridization (Table S1). Estimation of genes expressed in each tissues using MAS5 method showed that among the 46,857 gene models (TIGR V6.1), 31,451 of them can be detected in at least one of the analyzed tissues, 22,051 genes (70% of expressed genes) were expressed during seed development, and 20,405 and 18,401 genes were expressed during embryo and endosperm development respectively (Figure S1). The number of expressed genes in endosperm at 16 DAF was much less than that of embryo or endosperm at other developmental stages.

**Figure 1:**
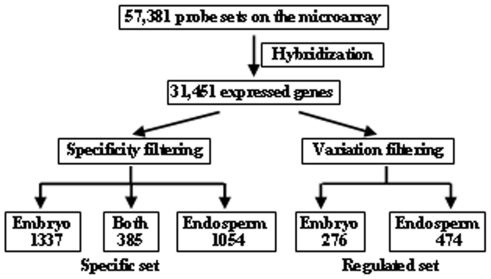
Analysis approaches of gene expression patterns in rice developing seeds. Work flow of data analysis was shown, genes of specific set and regulated set were analyzed respectively (See Materials and Methods section).

Limma package [24] was used to identify the genes predominantly expressed in embryo or endosperm, and ANOVA was performed to identify the regulated genes during seed development. As a result, 1,337 and 1,054 genes were identified to be predominantly expressed in embryo or endosperm respectively, and 385 genes were predominantly expressed in both embryo and endosperm (Figure 1). In addition, 276 and 474 genes were differentially expressed in embryo or endosperm respectively.

**Figure 2:**
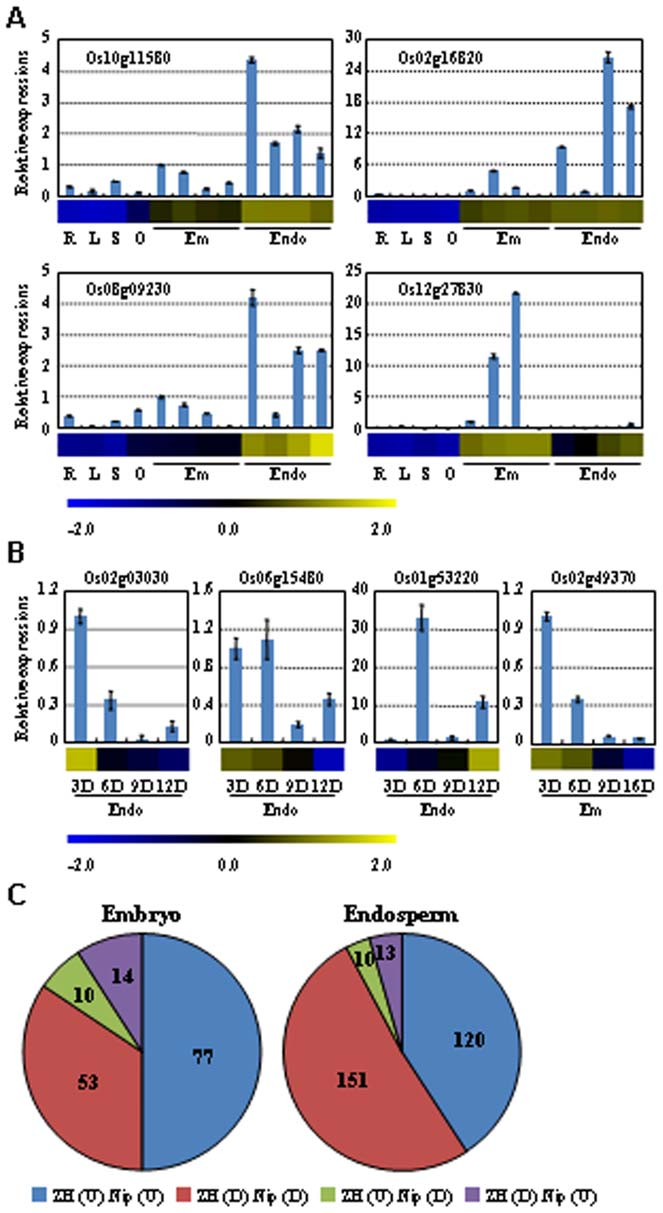
Corroboration of expression patterns of genes associated with seed development. The results by qRT-PCR analysis (box plot) are compared with data obtained from microarray hybridization (horizontal bars) to validate the predominantly expressed genes (A) and regulated genes (B). Expression of genes in root (R), leaf (L), seedling (S), ovary (O), embryo (Em) and endosperm (Endo) was analyzed and the value at 3 DAF in embryo was set as 1.0 for predominantly expressed genes (A). For the time course expression (B), the first time point was set as 1. Blue and yellow in cells reflect low or high expression levels, respectively, as indicated in the scale bar. The numbers of genes with different pattern in Zhonghua 11 (ZH) and Nipponbare (Nip) were calculated in embryo and endosperm (C). “U” or “D” in parenthesis indicates the genes are up-regulated or down-regulated. The data of Nip were obtained from Gene Expression Omnibus (GSE21494).

To validate the accuracy of expression patterns revealed by microarray hybridization, expression patterns of predominantly expressed and regulated genes was analyzed by real-time quantitative PCR (qRT-PCR) and the results indicated the consist expression patterns for most of the tested genes (Figure 2A, 2B), except a gene (Os01g53220, which is up-regulated during endosperm development). In addition, a microarray dataset (GSE21494 from Gene Expression Omnibus (GEO) at NCBI, [25]) including developing rice embryo and endosperm of japonica cultivar Nipponbare (similar as the ZH used in this study) was analyzed to compare with the data of our study. After filtering the low expression probes, the expression patterns of regulated genes during embryo or endosperm development showed high similarity in these two datasets (Figure S2). Detailed analysis indicated that among the 154 common regulated genes in embryo of two datasets, 84% of genes show same trends; and 92% of the 294 common genes in endosperm were similarly regulated (Figure 2C). These results indicated the high repeatability and reliability of the microarray data in this study.

**Figure 3:**
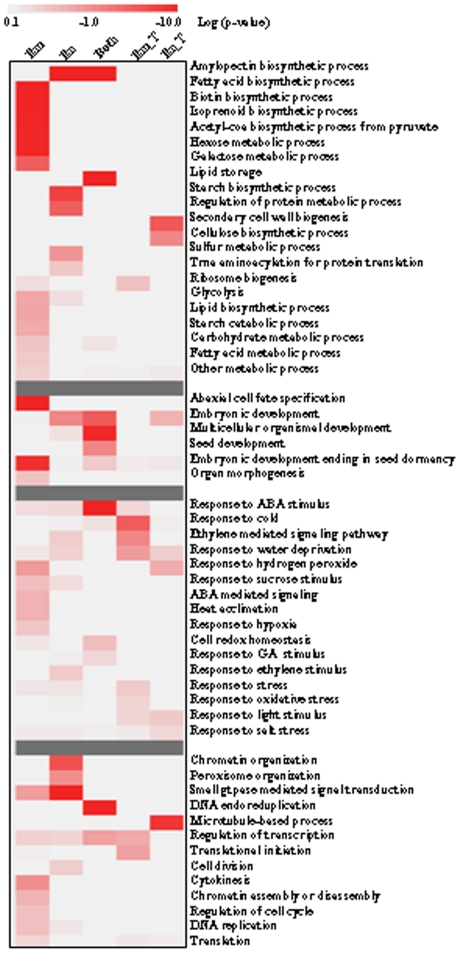
GO category enrichment of seed development-associated genes. The enrichment of process categories associated with metabolism, development, hormone signaling and cellular regulation were shown. “Em” or “En” indicates genes that are predominantly expressed in embryo or endosperm, respectively; “Both” indicates genes that are predominantly expressed in both embryo and endosperm; “Em_T” or “En_T” indicates genes that are regulated in time during embryo or endosperm development, respectively. Chi-square test was performed to calculate the p-values, which were log10 transformed. As indicated in the scale bar, the highly enriched GO categories were in red color.

Analysis of the biological processes of genes associated with seed development showed that many known genes involved in development, organ morphogenesis were highly expressed. In addition, genes associated with metabolism regulation, hormone response and cellular organization were also highly expressed in rice seed (Figure 3). Genes related to starch and protein metabolisms were enriched in endosperm and those involved in fatty acid biosynthetic and hexose metabolism were enriched in embryo. The expressions of genes involved in cellulose biosynthesis were altered during endosperm development (Figure 3). Interestingly, genes involved in starch synthesis were highly expressed in endosperm or the whole seed (Figure S3A) and those of starch degradation and fatty acid synthesis were highly expressed in embryo only (Figure S3B). In addition, genes involved in cellular processes including cell division, chromatin assembly or disassembly, microtubule-based process, transcription regulation were induced or suppressed during seed development (Figure 3).

### Hormone metabolism and signaling during rice seed development

Plant hormones are crucial regulators of growth and development, especially the processes of cell proliferation and differentiation [26]. Among the genes involved in hormone signaling, those responsive to abscisic acid (ABA) were highly expressed in both embryo and endosperm, and those involved in ethylene signaling were induced during embryo development (Figure 3).

**Figure 4:**
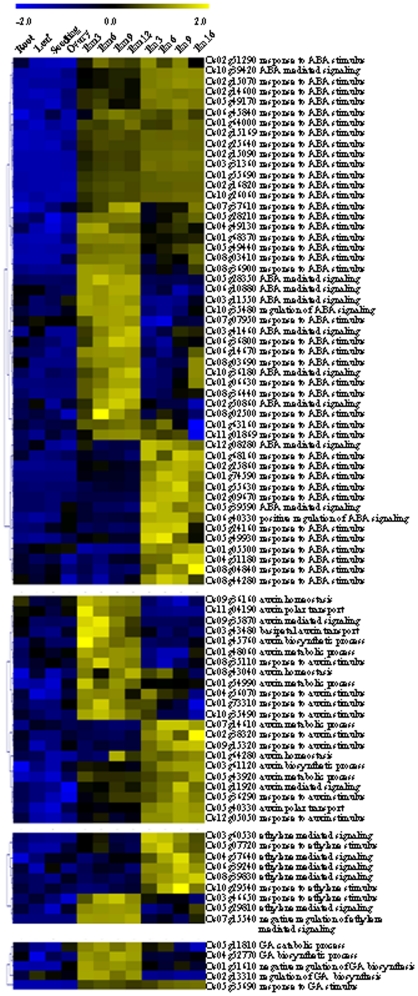
Expression patterns of genes involved in hormone biosynthesis and signaling, which are highly expressed in rice seeds. The expression data were normalized using Z score and genes involved in ABA, auxin, ethylene and gibberellin pathways were shown. The scale bar was shown at the top the figure.

Interestingly, most of the hormone-related genes that are regulated or predominantly expressed in seed were involved in ABA response (40 genes) and signaling (11 genes) (Figure 4), which is consistent with the crucial roles of ABA during seed maturation [27]. Among these genes, 23 and 14 genes were highly expressed in embryo or endosperm and other 14 genes were highly expressed in the whole seed.

Genes associated with auxin was less than that associated with ABA, and are involved in diverse functions including auxin biosynthetic and metabolic processes, polar auxin transport, auxin homeostasis and auxin-mediated signaling pathway. Six genes involved in ethylene-mediated signaling and 3 genes response to ethylene stimulus were highly expressed in seed. Four genes involved in the regulation of gibberellin metabolism were highly expressed in embryo, one of which encodes gibberellin 2-beta-dioxygenase 1 (Os05g11810). GASR6 (Os05g35690, gibberellin-regulated GASA/GAST/Snakin family protein precursor, which is responsive to gibberellin stimulus) was highly expressed in both embryo and endosperm (Figure 4).

Among the 12 hormone-related genes that were up-regulated during embryo development (Figure S4A), 5 were associated with ABA stimulus and 4 were associated with ethylene-mediated signaling, 2 were responsive to auxin stimulus, and one was involved in polar auxin transport (coding for N-1-naphthylphthalamic acid-binding protein). A gene (Os09g27820) encoding 1-aminocyclopropane-1-carboxylate oxidase 1, a rate-limiting enzyme in ethylene biosynthesis, was down-regulated during embryo development (Figure S4A). Among the 9 hormone-related genes associated with endosperm development, 6 genes were induced and 4 of them were involved in auxin response (Figure S4B).

### Expression patterns of ABA- and GA-biosynthesis genes

**Figure 5:**
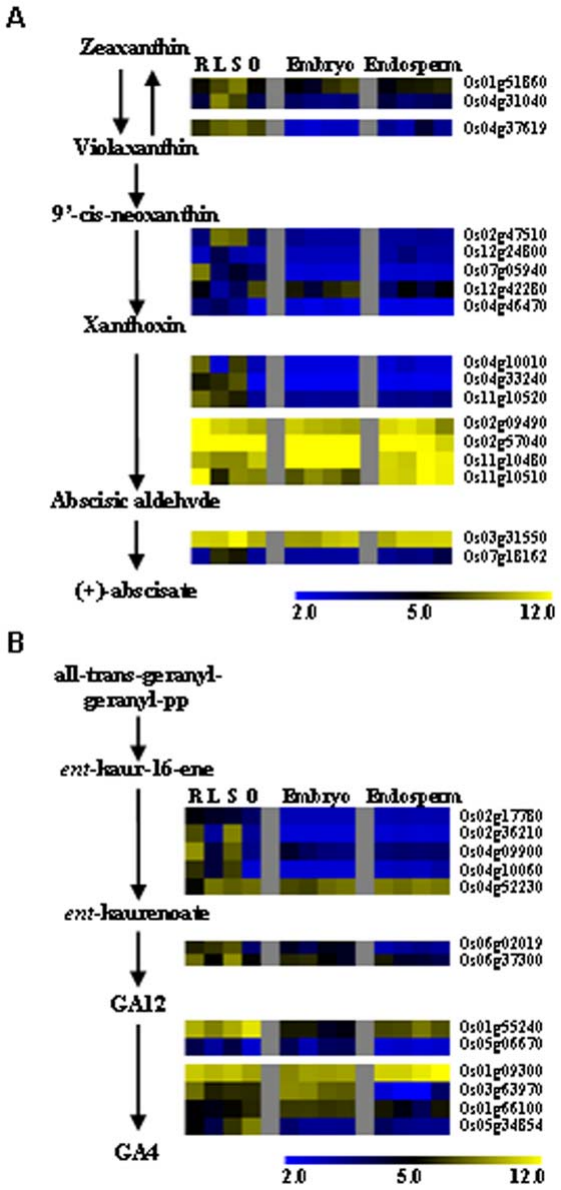
Expression patterns of genes involved in ABA and gibberellin biosynthesis during rice seed development. The ABA (A) and GA (B) biosynthesis pathways were from PlantCyc (www.plantcyc.org) and the expressions of relevant genes were log2 transformed. The expression of genes in root (R), leaf (L), seedling (S), ovary (O), embryo and endosperm were shown. The bar represented the scale of relative expression levels.

To further characterize the correlation of hormone biosynthesis and rice seed development, expression patterns of genes involved in hormone biosynthesis were analyzed. 9-cis-epoxycarotenoid dioxygenase (NCED) functions as a key enzyme in ABA biosynthesis (from 9′-cis-neoxanthin to xanthoxin) and the expression of candidate genes encoding NCED showed different patterns. Os12g42280 was highly expressed in rice seed and up-regulated during embryo development, while Os07g05940 was predominantly expressed in root and Os02g47510 was highly expressed in leaf and seedling, suggesting that Os12g42280 may function as a rate-limiting enzyme of ABA biosynthesis in rice seed. Besides to NCED, a gene (Os01g51860) encoding violaxanthin de-epoxidase that functions in the violaxanthin cycle at the upstream of ABA biosynthesis pathway [28], was induced during embryo and endosperm development. However, most of the genes involved in ABA biosynthesis were predominantly expressed in leaf or seedling, or in different tissues at similar level (Figure 5A). Differential expressions of genes encoding key enzymes of ABA biosynthesis at specific stages of seed development suggested the stringent control of ABA function at the synthetic level in rice seed.

Gibberellin 20 oxidase (GA20ox) catalyzes GA12 to GA4 and is the key enzyme in gibberellin biosynthesis. Among the candidate genes encoding GA20ox, Os01g09300 is highly expressed in many tissues and Os03g63970 is predominantly expressed in rice embryo and slightly repressed during embryo development. Among the genes encoding *ent*-kaurene synthase and *ent*-kaurene oxidase, Os04g09900 is down-regulated during embryo development and Os06g37300 is down-regulated during embryo and endosperm development (Figure 5B). These results are consistent with the crucial roles of GA at the early stages of seed development.

### Identification of transcription factors associated with seed development

The expression patterns of transcription factors (TFs) coding genes were surveyed using the annotations from two rice TF databases: DRTF [29] and RiceTFDB [30]. Among the 2,346 TF genes in rice genome, 2,231 genes were present on GeneChip® and 1,645 TF genes were expressed in rice seed, 1,356 and 1,131 TF genes were expressed in embryo or endosperm respectively. Further analysis indicated that 85 and 74 TFs were specifically expressed in embryo or endosperm respectively, and 32 TFs were highly expressed in both embryo and endosperm, 27 and 32 TFs were up- or down-regulated during embryo and endosperm development respectively (Table S2). These TFs fell into several families including bZIP, CCAAT, PHD, AP2/EREBP, bHLH, and ABI3/VP1, and some *Arabidopsis* homologs have been reported to regulate seed development (Table 1). Interestingly, many of them were also reported being involved in hormone signaling [31]. Chi-square test analysis showed that members of bZIP, CCAAT, PHD, ABI3/VP1, GRF families were enriched in genes associated with seed development, and CCAAT family members were highly expressed in both embryo and endosperm, while ABI3/VP1 family members were predominantly expressed in embryo (Table S2).

**Table 1:** Functions of conserved seed development-associated transcription factors.

Gene	TF Family	Homolog	Functions of homolog in Arabidopsis
High in embryo			
Os01g19970	MYB	MYB12	Flavonoid biosynthesis
Os01g48060	ARF	ETT	Auxin signaling
Os01g51610	ABI3/VP1	**FUS3**	Embryo development, ABA signaling
Os01g52680	MADS	AGL8	Fruit development
Os02g13310	HB	ATH1	Shoot apical meristem
Os02g42950	YABBY	AFO	Abaxial cell type specification
Os06g10880	bZIP	ABF1	ABA signaling
Os06g14670	MYB	MYB43	Secondary cell wall synthesis
Os07g15540	Orphans	EIN4	Ethylene signaling
Os08g06370	G2-like	KAN2	Polarity regualtion
Os08g36700	HSF	HSFB4	Asymmetric stem cell division
Os08g42600	RB	RBR1	Cell cycle, seed development
Os09g35870	AUX/IAA	AXR3	Auxin signaling
Os11g03540	AP2/EREBP	WRI1	Fatty acid synthesis
Os11g19060	AP2/EREBP	BBM	Embryo development
Os11g40100	GIF	GIF2	Organ size control
High in endosperm			
Os05g49930	GRAS	GAI	GA signaling
Os05g39590	AP2/EREBP	ABI4	ABA signaling
Os05g41070	bZIP	AREB3	ABA signaling
Os08g39830	EIL	EIL3	Ethylene signaling
Os02g28580	Orphans	MEE46	Endosperm development
Os11g31360	NAC	NAM	Regulate embryo
Os02g34320	bHLH	RGE1	Expressed in endosperm, regulate embryo
Os01g62310	HB	WOX2	Embryo development
Os05g43380	CPP	TSO1	Regulation of meristem organization
High in embryo and endosperm			
Os02g49410	CCAAT	**LEC1**	Embryo development
Os01g68370	ABI3/VP1	ABI3	Seed development, ABA signaling
Os01g64000	bZIP	ABI5	Seed development, ABA signaling
Os08g40030	NAC	CUC3	Meristem initiation
Os06g42630	ABI3/VP1	**B3 family**	Seed specific expression
Induced or suppressed in embryo			
Os02g49370	CCAAT	**NF-YB6**	Seed specific expression
Os02g52780	bZIP	ABF2	ABA signaling
Os03g20780	EIL	EIN3	Ethylene signaling
Induced or suppressed in endosperm			
Os03g57190	TCP	TCP2	Cell differentiation, leaf morphogenesis
Os02g06910	ARF	ARF6	Response to auxin stimulus
Os01g57110	SNF2	EDA16	Embryo sac development
Os12g40900	AUX/IAA	IAA3	Regulates multiple auxin responses

The LOC prefixes of all TIGR locus identifiers were removed for convenience. TFs in bold are seed-specific TFs in *Arabidopsis*.

**Figure 6:**
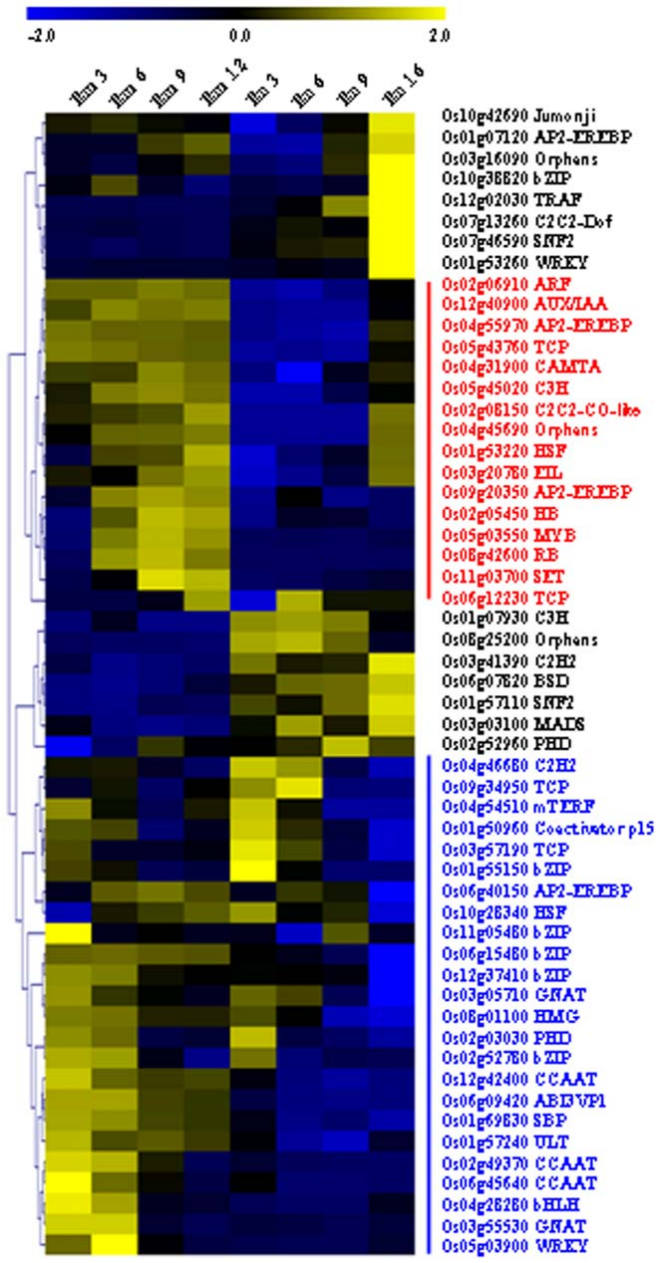
Expression patterns of transcription factor encoding genes during seed development. The expressions were normalized and the red or blue lines (words) indicate the up-regulated or down-regulated genes, respectively, during seed development. The scale bar was shown at the top the figure.

Gene clustering analysis of regulated TF genes revealed the similar regulation trends of TF genes in embryo and endosperm (Figure 6) and most of them were down-regulated during both embryo and endosperm development (blue line, Figure 6), same for up-regulated genes (red line, Figure 6). Eight TFs were highly expressed in endosperm at 16 DAF (Figure 6) and some of which had been reported to play roles in seed maturation. Three CCAAT-binding proteins, each of them belongs to three subfamilies (OsHAP2F/OsHAP3E/OsHAP5B) that might form a heterotrimeric complex to regulate transcription [32], were down-regulated during embryo development, indicating the important function of this complex during embryo development.

### Transcriptional networks controlling seed development

**Figure 7:**
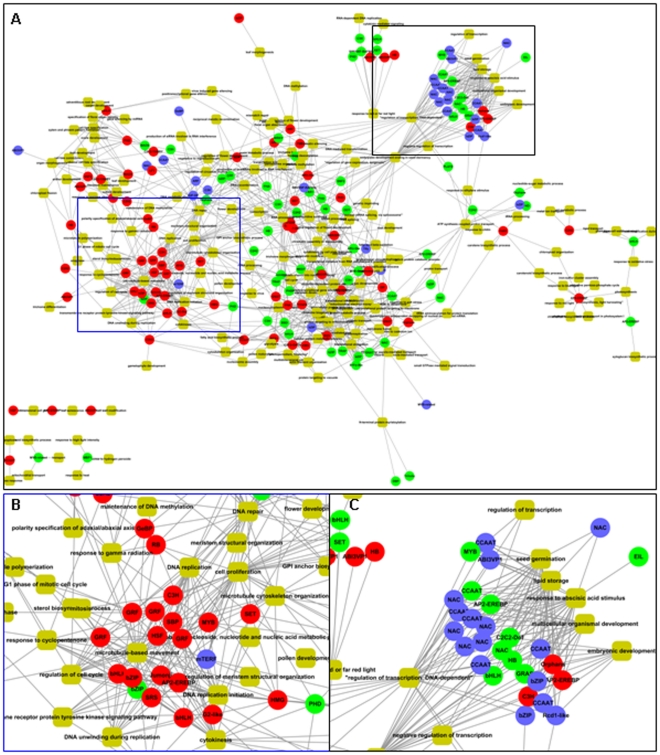
Transcriptional co-expression network involved in seed development. The relationship of transcription factors (circle) and the associated functions (yellow round rectangle) were shown as overview (A) and two modules predominantly expressed in embryo (B) and endosperm (C) respectively, were shown in detail. The circles with red, green and blue colors represent the transcription factors expressed in embryo, endosperm, or both embryo and endosperm.

To elucidate the functions of individual TFs in seed development, gene co-expression network of these TFs was constructed using public available microarray dataset from GEO at NCBI. Only the microarrays of Affymetrix platform (GPL2025 in GEO) were used for analysis (refer to Materials and Methods section). After normalization, Pearson correlation coefficients (PCCs) between TFs and expressed genes in the whole genome were calculated and genes co-regulated with TFs were used for gene ontology (GO) annotation and network construction (Figure 7). Biological processes of these TFs were inferred during the analysis. The results revealed that TFs expressed in embryo, including 4 of GRF family and members of bHLH, bZIP, SBP families, were associated with genes involved in the regulation of cellular processes, such as DNA replication, cell proliferation, and cell cycle regulation (Figure 7B), while TFs expressed in endosperm and whole seed were mostly associated with genes involved in the regulation of nutrients storage and response to ABA. In addition, TFs associated with embryo development were highly expressed in both embryo and endosperm (Figure 7C), including the members of C2H2, HB, bHLH and AP2/EREBP families.

Further analysis of the *cis*-elements in promoters of genes associated with seed development revealed that among the known *cis*-elements from PLACE [33], many *cis*-elements involved in the regulation of glutelin gene expression and ABA signaling were enriched in the promoters of genes predominantly expressed in endosperm and whole seed (Table S3). Several *cis*-elements associated with sugar and ethylene signalling were enriched in the promoters of genes in embryo, and an auxin responsive *cis*-element was enriched in genes regulated during embryo development (Table S3). In addition, *cis*-elements fell into bZIP, AP2 and B3 types were detected, which was consistent with the observation that members of these families were involved in seed development (Table S2).

In addition, many protein kinase genes were differentially expressed during seed development and/or predominantly expressed in seed (Table S4), of which two cell division cycle controlling proteins were highly expressed in both embryo and endosperm. Carbon catabolite derepressing protein kinase, also known as Sucrose Nonfermenting-1-Related Protein Kinase (SnRK), plays roles in carbohydrate metabolism regulation and two genes encoding SnRK were predominantly expressed in endosperm. Many receptor-like protein kinases (RLKs), calcium-dependent protein kinases and casein kinases were predominantly expressed in endosperm, some of which were regulated during endosperm development (Table S4). These protein kinases may cooperate with the transcriptional networks to refine the regulation of genes during seed development.

### Rice seeds are sensitive to low temperature at early stages of development and multiple biological processes are involved in the low temperature responses

**Figure 8:**
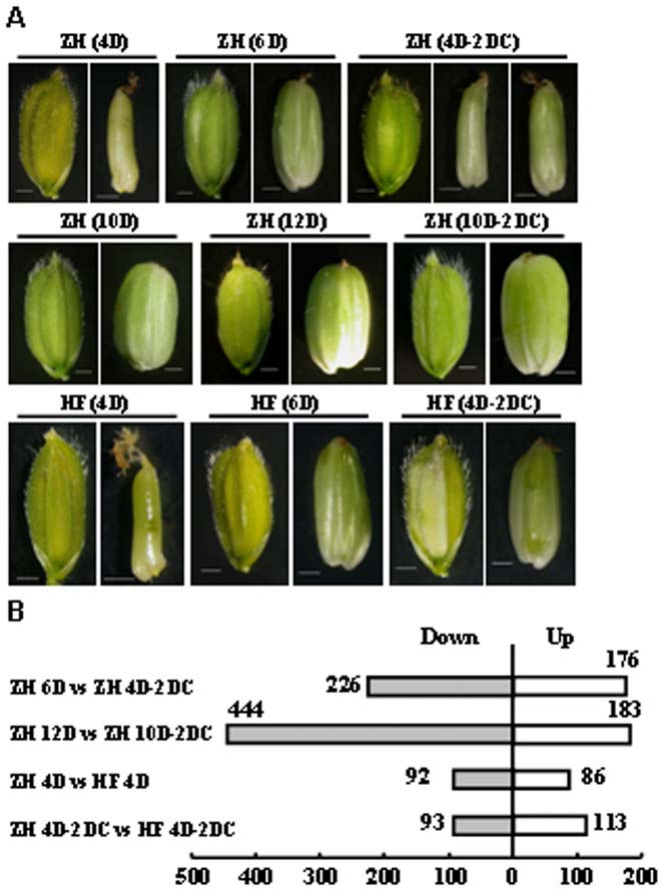
Regulated genes in chilling response of rice seeds. A. Low temperature treated rice seed of Zhonghua 11 (ZH) and Hanfeng (HF). Developing seeds of ZH and HF at 4 DAF were treated at 14°C for 2 days (4 D-2 DC) and seeds developed for 6 days (6 D) were used as control. Developing seeds of ZH at 10 DAF were treated at 14°C for 2 days (10 D-2 DC) and ZH seeds developed for 12 days (12 D) were used as control. Appearance of glumes and grains were shown under each treatment. Bar = 1 mm. B. Numbers of genes regulated by low temperature. ZH or HF represents the Zhonghua 11 or Hanfeng variety. “C” indicates the low temperature treatment; “D” indicates days after fertilization. Rice plants at 4 or 10 days after fertilization were treated with low temperature (14°C) for 2 days (ZH 4D-2DC, ZH 10D-2DC and HF 4D-2DC), with corresponding controls growing at normal temperature (ZH6 D, ZH 12D, HF6D). In each comparison, “Up” indicates number of genes higher expressed in later group, whereas “Down” indicates number of genes lower expressed in later group.

Rice is sensitive to low temperature and rice leaves rolled accompanying with the increase of relative ion leakage in leaves when rice seedlings were treated at 6°C for 24 h, which could fully expand after 24 h of recovery [34]. Being a cold-sensitive cultivar, the development of ZH seed was delayed when treated at 14°C for two days at 4 DAF (Figure 8A). However, when treated at 10 DAF, there was no obvious difference at the appearance. This indicated that the development of rice seed at early stage was more sensitive to chilling temperature. In contrast with ZH, cultivar Hanfeng (HF) showed chilling tolerance at the early stage of seed development and similar seed appearance were observed with or without low temperature treatment (Figure 8A).

To identify genes involved in the cold resistance, total RNAs of both ZH and HF seeds before and after low temperature treatment at two stages (4 or 10 DAF) were extracted, following by microarray hybridizations. Totally eight samples were collected and each sample has two biological replicates. Among the genes regulated by low temperature in ZH, the numbers of down-regulated genes were much more than the up-regulated ones (Figure 8B), which was different from the observation in *Arabidopsis* seedling [35]. To confirm whether the difference was due to the species, the raw data of low temperature response transcriptome of rice seedling (GSE6901) was downloaded from GEO at NCBI [36] and analysis showed that 912 or 826 genes were up- or down-regulated by low temperature in rice seedling respectively (same analysis pipelines as in rice seed were applied), which was consistent with that in *Arabidopsis* seedling but different from gene regulation in rice seed.

Comparison analysis showed that more genes are differentially expressed genes in ZH and HF under low temperature than at normal temperature (Figure 8B), indicating more genes are responsive to low temperature rather than constitutive expressed. Further analysis of the biological processes of the low temperature regulated genes showed that genes in cold acclimation process were significantly up-regulated at both two stages (Table 2) and genes involved in heat response and signal transduction components of plant hormones were also regulated by low temperature. Genes related to auxin and ABA signaling were up-regulated, whereas those of brassinosteroids biosynthesis and cytokinin signaling were down-regulated by low temperature at 4 DAF or 10 DAF respectively, suggesting the complex regulation by hormones in response to low temperature during seed development.

**Table 2:** Genes associated with different biological processes regulated by low temperature in Zhonghua 11.

GO ID	GO terms	Up_4	Do_4	Up_10	Do_10
Temperature response					
GO:0009408	Response to heat	4.500	7.000	45.600	-
GO:0010286	Heat acclimation	1.780	1.730	26.400	-
GO:0009631	Cold acclimation	2.820	-	2.880	-
GO:0009266	Response to temperature stimulus	2.550	-	2.610	-
Hormone					
GO:0009851	Auxin biosynthetic process	3.560	-	-	1.410
GO:0009734	Auxin mediated signaling pathway	-	-	2.280	-
GO:0009733	Response to auxin stimulus	1.569	-	-	-
GO:0009737	Response to ABA stimulus	-	-	3.500	-
GO:0009788	Negative regulation of ABA mediated signaling	3.147	-	-	-
GO:0009735	Response to cytokinin stimulus	-	-	-	2.640
GO:0016132	Brassinosteroid biosynthetic process	-	4.330	-	-
Chloroplast organization					
GO:0010020	Chloroplast fission	4.821	-	4.931	-
GO:0009658	Chloroplast organization and biogenesis	4.510	-	4.623	-
GO:0045037	Protein import into chloroplast stroma	3.809	-	3.896	1.515
Biosynthetic and metabolic process					
GO:0016117	Carotenoid biosynthetic process	8.264	-	2.283	-
GO:0006807	Nitrogen compound metabolic process	-	3.481	-	5.233
GO:0009718	Anthocyanin biosynthetic process	-	3.725	-	-
GO:0009089	Lysine biosynthetic process via diaminopimelate	-	-	4.189	-
GO:0005986	Sucrose biosynthetic process	-	-	3.043	-
GO:0016051	Carbohydrate biosynthetic process	-	-	-	2.831
GO:0005975	Carbohydrate metabolic process	-	-	-	2.643
Cellular process					
GO:0006298	Mismatch repair	25.996	-	-	-
GO:0006281	DNA repair	-	-	-	8.891
GO:0006974	Response to DNA damage stimulus	4.428	-	-	-
GO:0000911	Cytokinesis by cell plate formation	2.678	-	-	3.853
GO:0006260	DNA replication	2.645	-	-	3.664
GO:0007018	Microtubule-based movement	-	-	-	3.043
GO:0016049	Cell growth	-	-	-	2.551
GO:0006355	Regulation of transcription	-	1.813	-	-

“Up_4” or “Up_10” indicated the up-regulated genes by low temperature at 4 or 10 days after fertilization; “Do_4” or “Do_10” indicated the down-regulated genes by low temperature at 4 or 10 days after fertilization. Chi-square test was performed to test the enrichment of each GO term. The data indicates −log10 (P value). “-” indicated the corresponding genes were not enriched for the GO term.

Photosynthetic apparatus, especially the electron transport chain, is one of the main targets under low temperature stress [37]. Indeed, genes in chloroplast organization and chloroplast fission were up-regulated under low temperature in seed. Furthermore, genes of lysine and sucrose biosynthesis were induced to provide numerous compatible solutes for cellular water homeostasis during low temperature stress in rice seed (Table 2), and genes involved in ion transport were up-regulated by low temperature, which will maintain the ion homeostasis.

### Expression patterns of genes in chilling response and tolerance

**Figure 9:**
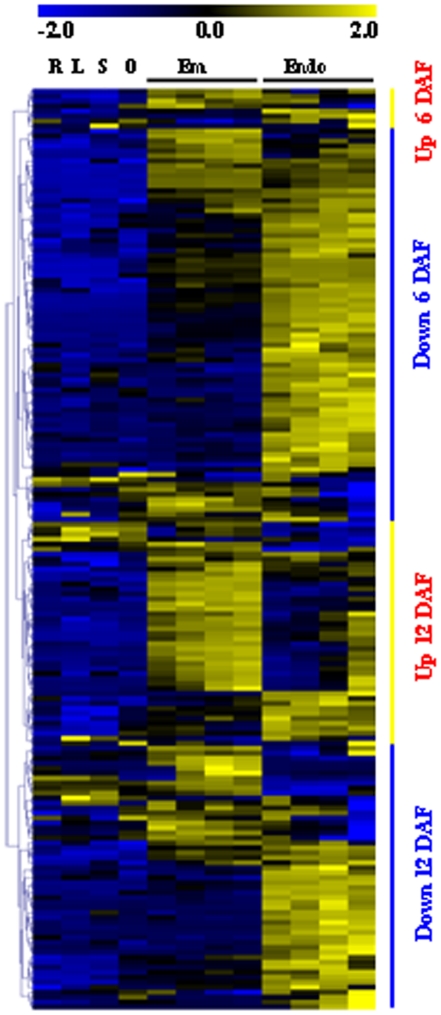
Expression patterns of seed development associated genes regulated by low temperature. The vertical bar indicated the pattern of genes regulated by cold in Zhonghua 11(ZH) seeds. Heat map showed the expression pattern during embryo (Em) and endosperm (Endo) development and in root (R), leaf (L), seedling (S) and ovary (O). The data were normalized using Z score as indicated in the scale bar.

To characterize the functions of genes regulated by low temperature in rice seed, the expression patterns of these genes at normal temperature were analyzed. Most of the regulated genes by low temperature at early stage (4–6 DAF) were down-regulated, whereas the numbers of up-regulated and down-regulated genes were similar at later stage (10–12 DAF, Figure 9). Some seed development associated TFs were regulated under low temperature (Table S5), indicating the close link between these two processes and involvement of multiple processes in low temperature responses. For example, MBF1c (multi-protein bridging factor 1c, involved in temperature response [38]) is predominantly expressed in endosperm and down-regulated by low temperature. RRTF1 (redox responsive transcription factor 1) and HSFB2B (class B-heat shock factor), and two TFs, TOC1 [39] and PCL1 [40], function in circadian rhythm regulation and are down-regulated under low temperature.

**Figure 10:**
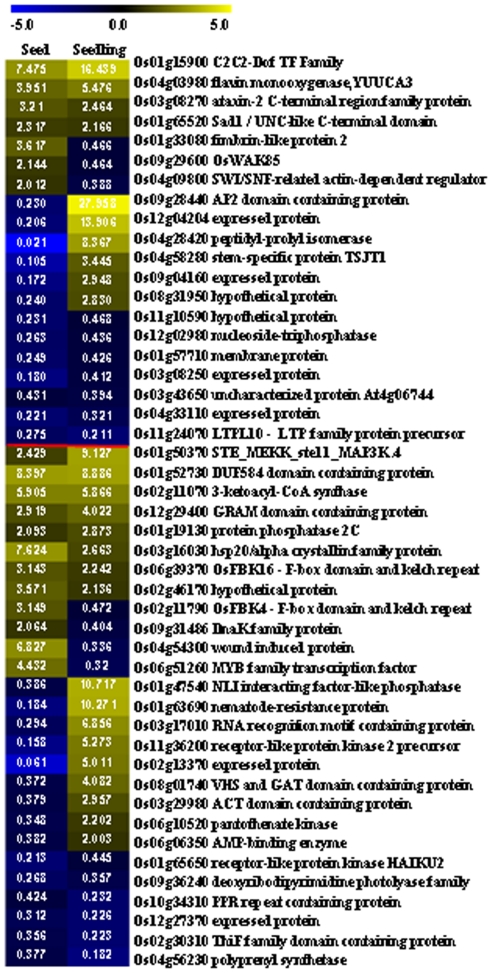
Common genes regulated by low temperature in seed and seedling. Genes above red line are regulated at 6 DAF and genes under red line are regulated at 12 DAF. The numbers indicate the ratios of gene expressions regulated by low temperature, and the color represents the log2 transformed ratio as indicated in the scale bar.

Comparison of the expression patterns of genes regulated by low temperature in seed and seedling showed that among the 1,033 and 1,738 regulated genes, only 48 genes were regulated in both seed and seedling, and only half of them were in the same trend (Figure 10). An AP2 TF was induced by low temperature in seedling for 28 fold, whereas down-regulated by low temperature in seed for >4 fold. A C2C2-DOF TF was induced by low temperature for 7 and 16 fold in seed and seedling respectively. These suggested that genes involved in low temperature response may function through same or different mechanisms in seed and seedling.

Regarding the metabolic processes, genes related to compatible solutes metabolism (such as lipids, sucrose, and aromatic amino acid) and phospholipid biosynthesis were highly expressed in HF than in ZH (Table S6). The higher expressions of genes in reactive oxygen species (ROS) metabolic process in ZH suggested that the sensitivity of ZH to low temperature at early stage may due to the higher ROS levels. In addition, genes involved in posttranscriptional gene silencing and protein modification (glycosylation, dephosphorylation and ubiquitination) were also altered, suggesting that the posttranscriptional regulation were also involved in low temperature tolerance.

## Discussion

### Complex gene regulation network in rice embryo and endosperm

Although some transcriptome analyses of rice seeds have been performed, most of the studies were done at single time points of multiple genotypes/cultivars [15]–[19] or at differential developmental stages of entire seeds [14]. We here reported the analysis of gene profile in rice embryo or endosperm at four developmental stages, providing the information of transcriptional dynamics in rice embryo and endosperm.

With a similar proportion of genes compared to *Arabidopsis* [41], around 70% of genes are expressed during rice seed development, indicating some conserve mechanisms in evolution between these two model plants. Among the genes expressed in rice seed, genes expressed in endosperm, especially at 16 days after fertilization (DAF), are less than those in embryo (Figure S1), which might due to the different cell types of these two tissues. During embryo development, several types of cells undergo differentiation and possess higher activities, whereas the cell types in endosperm are relatively simpler and programmed cell death (PCD) is initiated during endosperm development, resulting in the accumulation of a spectrum of nuclease activities to repress the expressions of genes in endosperm [42].

Genes involved in diverse metabolism pathways are highly expressed in rice seed, being consistent with the observation that the expression of these genes is coordinately controlled in a synchronized manner during grain filling [14]. Genes in starch metabolism and fatty acid synthesis are highly expressed in endosperm or embryo respectively, which is consistent with the location where they are mostly synthesized. Cellularization of endosperm is an essential process in endosperm development, during which cell wall biogenesis and deposition and microtubular cytoskeletal apparatus organization play important roles [43], which are supported by our results that genes in cell wall biogenesis process and microtubule-based process are enriched during endosperm development. In rice embryo and endosperm, different cell types have diverse physiological functions and cell fate specification is believed to be determined by positional signaling [43]. Further study of transcriptomes in different cell types using specific strategies, such as laser capture microdissection (LCM) [44], will provide more information of the regulation in seed development.

### Crucial roles of hormone in seed development

Highly expressed hormone-related genes are mainly involved in ABA response, suggesting the important roles of ABA in seed development [27]. It is interesting to notice that none of ABA-biosynthesis genes are predominantly expressed in rice seed (Figure 4), indicating the subtle spatial and temporal regulation of ABA biosynthesis in rice seed and ABA may be synthesized in seed only at certain developmental stages.

Genes related to auxin biosynthetic and metabolic processes, polar auxin transport, homeostasis and auxin-mediated signaling, are highly expressed in rice seed, being consistent with the high accumulation of IAA in rice seed [45] and the crucial role of auxin in embryo axis formation and embryo development. Recent studies showed that PIN1-mediated auxin transport was related to cellular differentiation during maize embryogenesis and endosperm development [46] and essential role of OsARF1 in rice seed development [47]. Altered auxin signaling in seeds may cause abnormal development, proposing an “autonomous” mechanism of auxin signaling in seeds.

Ethylene inhibits ABA signaling during seed development [48] and our results also showed that a gene coding the rate-limiting enzyme in ethylene biosynthesis is down-regulated during embryo development (Figure S4A), being consistent with the enhanced ABA signaling in rice embryo. GAs are required for plant seed development and GA-stimulated Arabidopsis (GASA) genes are induced by GAs [49]. Our results indicate that genes encoding gibberellin 2-beta-dioxygenase 1 is highly expressed in embryo and GASR6 (Os05g35690) is highly expressed in both embryo and endosperm, suggesting that these two genes may be the key factors regulating GAs biosynthesis in rice seed. Two genes encoding *ent*-kaurene synthase and *ent*-kaurene oxidase are down-regulated during seed development, further indicating the function of GAs at the early stages of seed development.

As a whole, our data indicate the diverse patterns of hormone signaling in rice seed development. The fact that genes in these hormone signaling pathways are regulated in different trends or stages suggested that hormones' impact may cover different processes in rice seed development, independently or interactively.

### Crucial roles of transcriptional regulation and TFs during seed development

191 TFs are predominantly expressed in rice seed and 59 TFs are up- or down-regulated during seed development, providing informative clues on studying the transcriptional regulation of rice seeds. TFs of bZIP, CCAAT, PHD, AP2/EREBP, bHLH, ABI3/VP1, and NAC families were predominantly expressed in seed, which is consistent with the studies that genes in most of these families are associated with seed development. bZIP TFs are expressed in aleurone and endosperm cells of developing rice seeds [50] and bind to alpha-globulin promoters to regulate the expression of genes encoding storage proteins [51]. bZIP TFs also bind to ABA-responsive elements (ABREs) to mediate the ABA-induced transcription [52]. Several CCAAT family members are highly expressed at early stages of embryo development and then down-regulated. As the encoded proteins may function through forming a heterotrimeric complex to regulate transcription [32], the coordinately regulation of members of this complex suggests its function in the early stages of embryo development.

Studies of the *Arabidopsis* homologs of seed development related TFs showed that most of them are with conserved functions (Table 1). TFs FUS3, BBM, and RBR1 are predominantly expressed in *Arabidopsis* embryo, which is same in rice embryo. A TF homolog to WRI1, which has been reported to regulate fatty acid synthesis, is identified in rice embryo, suggesting the conserved function of WRI1 in rice and *Arabidopsis*. RGE1 is highly expressed in *Arabidopsis* endosperm and involved in embryo development regulation [53], and the closest homolog of *Arabidopsis* RGE1, Os02g34320, is highly expressed in rice endosperm, indicating its conserved function. In addition, seed-specific TF gene set is enriched for known regulators of seed development, suggesting the TFs with unknown function in this set are also critical for seed development.

Several TFs are involved in both seed development and hormone singling. For example, an AP2/EREBP TF OsEBP-89, is temporally expressed in developing endosperm and is involved in ethylene-dependent seed maturation [31]. Presence of hormone-responsive elements in the promoters of seed-predominant genes (Table S3) highlights the roles of TFs as connexon between hormone signaling and seed development.

### Gene expression reprogramming in response to low temperature

Genes involved in cold acclimation process and heat response are both up-regulated under low temperature, which is consistent with the fact that many common elements such as heat shock proteins, active oxygen species, compatible solutes, membrane lipids and transcriptional factors are involved in both cold and heat stress response [54]. Genes in chloroplast organization are induced in Zhonghua 11 (ZH) and are expressed at a higher level in Hanfeng (HF) under low temperature, suggesting these genes may endow the more vibrant metabolism activity to cope with chilling stress. Enhanced expression of genes in pathways of stress-related hormones (ABA, ethylene and jasmonic acid) (Table 2), indicating the roles of these hormones in stimulating the stress responses in seeds through up-regulating the stress associated TFs.

The reprogramming of gene expression is of central role during low temperature response [22], and many TFs including MYB-related, PHD, HSF, C2H2 and AP2/EREBP families are regulated by low temperature and may involve in stress response and/or seed development. Among the seed-associated genes regulated by low temperature, most of them are with suppressed expression, indicating that seed development is delayed by low temperature through inhibition of the transcription of these genes at early stage. Indeed, all the low temperature regulated AP2/EREBP TFs are down-regulated in rice seed at 4 DAF, whereas most of low temperature regulated AP2/EREBP TFs are up-regulated in seedling of *Arabidopsis* [35] and rice. CBF/DREBs (AP2/EREBP family) play important roles in low temperature response and cold acclimation by regulating downstream genes like COR, KIN and LTI [22], suggesting that the CBF/DREB is differentially regulated in early seed and seedling, which is consistent with the observation that more genes are suppressed by low temperature in rice seeds whereas more genes are induced in rice and *Arabidopsis* seedlings. The transcriptional difference might be the consequence of two factors. The first is that seeds at early stage are more sensitive to chilling stress than seedlings (Figure 8A). The developments of seeds are arrested while there is no visible difference in leaves after low temperature treatment. The second is that the targets of the CBF/DREB regulations are different in seed and seedling. The targets of these TFs in rice seed may mostly involve in developmental regulation and down-regulation of these genes causes the inhibition of seed development, while the induction of target genes in seedling may endow tolerance ability to chilling stresses.

Several pathways are differentially regulated by low temperature in ZH and HF, which may contribute to the chilling tolerance of HF. Genes associated with lipid metabolism and glycolysis are highly expressed in HF at both normal and low temperature, whereas genes in sucrose metabolism and aromatic amino acid family biosynthetic processes are only highly expressed after low temperature treatment. It has been reported that cold tolerance is positively correlated with the expression level of genes in carbohydrate, amino acid and secondary metabolism [55]. The chilling tolerance of HF cultivar may be caused, at least partially, by the higher level of carbohydrate metabolism. Second messenger molecules are involved in low temperature response in *Arabidopsis* [22] and our study showed that genes of phospholipid biosynthetic process are highly expressed in HF after low temperature treatment, whereas genes in phosphatidylinositol metabolic process are expressed at a lower level in HF. Phospholipid signaling plays important roles in regulating plant growth and development [56] and differential expression of genes in this pathway may contribute to the higher level of phospholipid molecules and activate genes in chilling response.

### Conclusions

Our systematic studies indicated that many factors, especially transcription factors and plant hormones, play central roles in the regulation of rice seed development and the response to low temperature, through regulation of multiple processes including chromatin assembly, cell division, cell fate specification, and organ morphogenesis. The identified TFs are valuable candidates for study of the seed development regulation. Our analysis showed that seed development is inhibited by low temperature through suppression of groups of genes at early stages, and the transcriptional response to chilling stress are of great difference between seeds and seedlings.

## Materials and Methods

### Plant material and growth conditions

Rice plants of two japonica cultivars, Zhonghua 11 (ZH) and Hanfeng (HF), were cultivated in a phytotron with a light (12 h, 28°C)/dark (12 h, 22°C) cycle. The embryo material was collected at 3, 6, 9 and 12 days after fertilization (DAF) and endosperm material was collected at 3, 6, 9 and 16 DAF. Leaves and roots were harvested at three-leaf stage (7-day-old) and seedlings were harvested at four-leaf stage (10-day-old). Ovary was collected as 0 DAF seed. Totally twelve samples were collected and each sample has two biological replicates.

For the low temperature treatment, rice plants of both ZH (cold-sensitive) and HF (cold-resistant) were transferred to 14°C for two days at 4 or 10 DAF respectively. The seeds before and after low temperature treatment were harvested to observe the appearance and to extract total RNAs. Totally eight samples were collected and each sample has two biological replicates. Expression data of these samples were compared to identify genes regulated by low temperature and genes involved in cold resistance.

### Microarray hybridization and data normalization

GeneChip® rice genome array (Affymetrix, Santa Clara, CA) was used in DNA microarray analysis. Total RNAs from embryo and endosperm of seeds, leaf, root, seedling, ovary and developing seeds with and without low temperature treatment were extracted using TRIzol reagent (Invitrogen Life Technologies) and 10 µg cRNA was used for hybridization. Washing, staining, and scanning were performed as described in the supplier's protocol. The hybridization signals were normalized using GC-Robust Multichip Average (GC-RMA) from Bioconductor (www.bioconductor.org) Suite of tools for the statistical package R (www.R-project.org). All the data can be accessed at GEO. The series GSE11966 includes data from rice seedling, leaf, root, embryo and endosperm at 6 DAF, GSE27856 includes data from rice ovary, embryo and endosperm of other developmental stages in ZH. The dataset from cold treatment experiments in ZH and HF seed is under GSE31077. Pearson correlation coefficients (PCCs) were carried out for each sample to check the replicate quality of microarray data.

### Data Statistical Analysis

The linear statistical model in Limma package [24] from Bioconductor was used to identify significant differentially expressed genes between two groups. ANOVA tool in TIGR MeV (version 4.0, http://www.tm4.org/mev.html) was used to identify genes differentially expressed in embryo and endosperm in time course. False discovery rates (FDRs) for various P value thresholds were later determined by described method [57]. To identify genes predominantly expressed in embryo and endosperm, the threshold was set as FDR adjusted P value<0.0005 and change ratio >2.0. For time course genes, the threshold was P value<0.001. For low temperature regulated genes, the threshold was set as P value<0.05 and change ratio >2.0. The raw data of cold response transcriptome of rice seedling was downloaded from GEO at NCBI (GSE6901), which included materials treated by low temperature (4°C). The analysis pipelines were same as performed in rice seed.

A recently published microarray dataset (GSE21494 in GEO, [25]) of Agilent platform (GPL6864) was compared with data of this study to validate the gene expression patterns. Firstly, the probes with low expression levels (<0) were filtered out and expression data of ten embryo and endosperm samples were then summarized by gene models in TIGR rice genome annotation (V6.1). The expression trends of genes regulated during embryo or endosperm developments were compared.

To annotate the functions of identified gene sets, the gene ontology information from gene ontology website (http://www.geneontology.org/), Rice Genome Annotation (http://rice.plantbiology.msu.edu/) and *Arabidopsis* annotation (ftp://ftp.arabidopsis.org/home/tair/Ontologies/Gene_Ontology/) were used. Chi-square test was performed to test the enrichment of GO terms in gene sets and the P values were log10 transformed for visualization using TIGR MeV.

### Data validation by real-time quantitative PCR (qRT-PCR)

Total RNAs were extracted using TRIzol reagent from different tissues same as that were collected for microarray hybridization. For each sample, cDNA was synthesized from 1 µg of total RNA using PrimeScript RT reagent Kit with gDNA Eraser (Perfect Real Time, TaKaRa DRR047A). qRT-PCR analysis was performed on Rotor-Gene RG-3000A (Corbett) using SYBR Green Real-time PCR Master Mix (TOYOBO QPK-201), with three biological replicates of each tissue. The values of threshold cycle (*C*_T_, the fractional cycle number at which the fluorescence passes the fixed threshold), were calculated by Rotor-Gene 6 software (Corbett Robotics, Australia) and converted into relative copy numbers using a standard curve. Gene *Actin1* (Os05g36290) was used as a reference gene as suggested by Caldana et al. [58] and the sequences of all the primers used in this study were listed in Table S7.

### Identification of enriched cis-elements at promoters of seed associated genes

Up to 3000 nt upstream sequence of each gene (from ATG) was extracted from the genome and known cis-elements were mapped onto the promoters using Plant Cis-acting Regulatory DNA Elements (PLACE). The ratio of each cis-element in a particular group was compared with that in the whole genome followed by performing Chi-square test. Because ∼200 known cis-elements were analyzed for each group, FDRs for various P value thresholds were determined. If a known cis-element was enriched with a low FDR adjusted P value (<0.05), this cis-element was annotated as enriched in the promoters of genes in a certain group.

### Annotation of gene functions using gene-coexpression analysis

Gene coexpression analysis was performed according to previous report [59]. In brief, Affymetrix hybridization array raw data of rice (GPL2025) was downloaded from GEO, and then normalized using GC-RMA method. The probe sets on the array with very low intensity through all the hybridizations were excluded (determined using MAS5 method). The Pearson correlation coefficients between probes representing each TF gene and other genes on the array were calculated. The GO terms were used to annotate the top 500 related genes of each TF gene. The relationships of transcription factors and GO terms were visualized using Cytoscape V2.8.0 [60].

## Supporting Information

Figure S1 **Numbers of genes expressed during seed development.** A probe is recorded as expressed when it is eliminated as “P” in two replicates. The numbers indicated the genes expressed in all the stages of embryo (Em) and endosperm (En) development.
(TIF)

Figure S2 **Validation of microarray data.** Expression pattern of genes regulated in embryo (A) and endosperm (B) of cultivar Nipponbare are compared with the trends in Zhonghua 11 (ZH). The left vertical bars indicate the individual gene is up-regulated (yellow) or down-regulated (blue) in ZH. “D” indicates days after fertilization (DAF). The data for heatmap were normalized by gene, and the value scale is shown at the bottom. The data of cultivar Nipponbare were from Gene Expression Omnibus (GSE21494).
(TIF)

Figure S3 **Genes involved in starch (A) and lipid (B) metabolism.** Genes are predominantly expressed in embryo (red), endosperm (blue) or both embryo and endosperm (black) are indicated. The annotations of genes are from MAPMAN (http://mapman.gabipd.org).
(TIF)

Figure S4 **Expression patterns of genes involved in hormone biosynthesis and signaling during embryo (A) or endosperm development (B).** The average data of two replicates are calculated, and normalized in Z-score. TIGR MeV was used for visualization.
(TIF)

Table S1 **Reproducibility of microarray data of each tissue.** “PCC” indicates Pearson correlation coefficient of data from the entire array. “ZH” or “HF” indicates the whole seed of Zhonghua 11 or Hanfeng respectively. “C” indicates the low temperature treatment (14°C) for 48 h, “D” indicates days after fertilization (DAF). Refer to Materials and Methods section for details.
(DOC)

Table S2 **Transcription factors associated with seed development.** The TF numbers of each family are shown. “Em” or “En” indicates the genes predominately expressed in embryo or endosperm, respectively. “Both” indicates that genes are predominately expressed in both embryo and endosperm; “Em_T” or “En_T” indicate that genes showing regulated expression pattern during embryo or endosperm development, respectively. “Total Number” indicates the total number of genes in each TF family in genome and “Sum of all TFs” indicates the number of all identified TF genes by hybridization. Chi-square test was performed to test the enrichment of each TF families, if the P value is less than 0.05, the number will be shown in bold.
(DOC)

Table S3 **Cis elements associated with seed development.** “Ra_G” or “Ra_L” indicate the ratio of cis element in the whole genome or in the gene lists, respectively. “FDR” indicates the FDR corrected P value.
(DOC)

Table S4 **Identified protein kinases associated with seed development.** “P value” is calculated from limma (for predominantly expressed genes) or by ANOVA analysis (for regulated genes).
(DOC)

Table S5 **Transcription factors (TFs) those are down-regulated in Zhonghua 11 seeds by low temperature at early stage.** “Type” indicates the expression pattern of TF genes during seed development, including En (endosperm), Em (embryo), Both (endosperm and embryo) and En_T (regulated expression pattern during endosperm development in time course).
(DOC)

Table S6 **Involved biological processes of genes with differential expression in Hanfeng variety.** ^a^Genes were highly expressed in Zhanghua 11 (ZH), ^b^genes were highly expressed in Hanfeng (HF). Chi-square test was performed to test the enrichment of each GO term. The data indicates −log10 (P value). “-” indicates that the genes are not enriched in the GO term.
(DOC)

Table S7 **Primers used in real time quantitative PCR (qRT-PCR) analysis.** “S” and “A” indicate the sense or antisense primer respectively.
(DOC)

## References

[1] Itoh J, Nonomura K, Ikeda K, Yamaki S, Inukai Y (2005). Rice plant development: from zygote to spikelet.. Plant Cell Physiol.

[2] Sato Y, Tamaoki M, Murakami T, Yamamoto N, Kano-Murakami Y (1996). Abnormal cell divisions in leaf primordia caused by the expression of the rice homeobox gene OSH1 lead to altered morphology of leaves in transgenic tobacco.. Mol Gen Genet.

[3] Agarwal P, Kapoor S, Tyagi AK (2011). Transcription factors regulating the progression of monocot and dicot seed development.. Bioessays.

[4] Kurata N, Miyoshi K, Nonomura K, Yamazaki Y, Ito Y (2005). Rice mutants and genes related to organ development, morphogenesis and physiological traits.. Plant Cell Physiol.

[5] Nagasaki H, Itoh J, Hayashi K, Hibara K, Satoh-Nagasawa N (2007). The small interfering RNA production pathway is required for shoot meristem initiation in rice.. Proc Natl Acad Sci USA.

[6] Sazuka T, Kamiya N, Nishimura T, Ohmae K, Sato Y (2009). A rice tryptophan deficient dwarf mutant, tdd1, contains a reduced level of indole acetic acid and develops abnormal flowers and organless embryos.. Plant J.

[7] Hattori T, Terada T, Hamasuna ST (1994). Sequence and functional analyses of the rice gene homologous to the maize Vp1.. Plant Mol Biol.

[8] Sun X, Shantharaj D, Kang X, Ni M (2010). Transcriptional and hormonal signaling control of Arabidopsis seed development.. Curr Opin Plant Biol.

[9] Ruuska SA, Girke T, Benning C, Ohlrogge JB (2002). Contrapuntal networks of gene expression during Arabidopsis seed filling.. Plant Cell.

[10] Lee JM, Williams ME, Tingey SV, Rafalski JA (2002). DNA array profiling of gene expression changes during maize embryo development.. Funct Integr Genomics.

[11] Liu X, Fu J, Gu D, Liu W, Liu T (2008). Genome-wide analysis of gene expression profiles during the kernel development of maize (*Zea mays* L.).. Genomics.

[12] Laudencia-Chingcuanco DL, Stamova BS, You FM, Lazo GR, Beckles D M (2007). Transcriptional profiling of wheat caryopsis development using cDNA microarrays.. Plant Mol Biol.

[13] Druka A, Muehlbauer G, Druka I, Caldo R, Baumann U (2006). An atlas of gene expression from seed to seed through barley development.. Funct Integr Genomics.

[14] Zhu T, Budworth P, Chen W, Provart N, Chang HS (2003). Transcriptional control of nutrient partitioning during rice grain filling.. Plant Biotechnol J.

[15] Ge X, Chen W, Song S, Wang W, Hu S (2008). Transcriptomic profiling of mature embryo from an elite super-hybrid rice LYP9 and its parental lines.. BMC Plant Biol.

[16] Tu Q, Dong H, Yao H, Fang Y, Dai C (2008). Global identification of significantly expressed genes in developing endosperm of rice by expression sequence tags and cDNA array approaches.. J Integr Plant Biol.

[17] Liu X, Guo T, Wan X, Wang H, Zhu M (2010). Transcriptome analysis of grain-filling caryopses reveals involvement of multiple regulatory pathways in chalky grain formation in rice.. BMC Genomics.

[18] Kim CK, Cho MA, Choi YH, Kim JA, Kim Y H (2011). Identification and characterization of seed-specific transcription factors regulating anthocyanin biosynthesis in black rice.. J Appl Genet.

[19] Venu R, Sreerekha M, Nobuta K, Belo A, Ning Y (2011). Deep sequencing reveals the complex and coordinated transcriptional regulation of genes related to grain quality in rice cultivars.. BMC Genomics.

[20] Duan K, Luo YH, Luo D, Xu ZH, Xue HW (2005). New insights into the complex and coordinated transcriptional regulation networks underlying rice seed development through cDNA chip-based analysis.. Plant Mol Biol.

[21] Satake T, Hayase H (1970). Male sterility caused by cooling treatment at the young microspore stage in rice plants. V. Estimation of pollen developmental stage and the most sensitive stage to coolness.. Proc Crop Sci Soc Jpn.

[22] Zhu J, Dong CH, Zhu JK (2007). Interplay between cold-responsive gene regulation, metabolism and RNA processing during plant cold acclimation.. Curr Opin Plant Biol.

[23] Agarwal M, Hao Y, Kapoor A, Dong CH, Fujii H (2006). A R2R3 type MYB transcription factor is involved in the cold regulation of CBF genes and in acquired freezing tolerance.. J Biol Chem.

[24] Wettenhall JM, Smyth GK (2004). LimmaGUI: a graphical user interface for linear modeling of microarray data.. Bioinformatics.

[25] Sato Y, Antonio B, Namiki N, Motoyama R, Sugimoto K (2011). Field transcriptome revealed critical developmental and physiological transitions involved in the expression of growth potential in japonica rice.. BMC Plant Biol.

[26] Long TA, Benfey PN (2006). Transcription factors and hormones: new insights into plant cell differentiation.. Curr Opin Cell Biol.

[27] Holdsworth MJ, Bentsink L, Soppe WJ (2008). Molecular networks regulating Arabidopsis seed maturation, after-ripening, dormancy and germination.. New Phytol.

[28] Hieber AD, Bugos RC, Yamamoto HY (2000). Plant lipocalins: violaxanthin de-epoxidase and zeaxanthin epoxidase.. Biochim Biophys Acta.

[29] Gao G, Zhong Y, Guo A, Zhu Q, Tang W (2006). DRTF: a database of rice transcription factors.. Bioinformatics.

[30] Riano-Pachon DM, Ruzicic S, Dreyer I, Mueller-Roeber B (2007). PlnTFDB: an integrative plant transcription factor database.. BMC Bioinformatics.

[31] Yang HJ, Shen H, Chen L, Xing YY, Wang ZY (2002). The OsEBP-89 gene of rice encodes a putative EREBP transcription factor and is temporally expressed in developing endosperm and intercalary meristem.. Plant Mol Biol.

[32] Thirumurugan T, Ito Y, Kubo T, Serizawa A, Kurata N (2008). Identification, characterization and interaction of HAP family genes in rice.. Mol Genet Genomics.

[33] Higo K, Ugawa Y, Iwamoto M, Korenaga T (1999). Plant cis-acting regulatory DNA elements (PLACE) database: 1999.. Nucleic Acids Res.

[34] Yan SP, Zhang QY, Tang ZC, Su WA, Sun WN (2006). Comparative proteomic analysis provides new insights into chilling stress responses in rice.. Mol Cell Proteomics.

[35] Lee BH, Henderson DA, Zhu JK (2005). The *Arabidopsis* cold-responsive transcriptome and its regulation by ICE1.. Plant Cell.

[36] Jain M, Nijhawan A, Arora R, Agarwal P, Ray S (2007). F-box proteins in rice. Genome-wide analysis, classification, temporal and spatial gene expression during panicle and seed development, and regulation by light and abiotic stress.. Plant Physiol.

[37] Ducruet JM, Peeva V, Havaux M (2007). Chlorophyll thermofluorescence and thermoluminescence as complementary tools for the study of temperature stress in plants.. Photosynth Res.

[38] Suzuki N, Bajad S, Shuman J, Shulaev V, Mittler R (2008). The transcriptional co-activator MBF1c is a key regulator of thermotolerance in *Arabidopsis thaliana*.. J Biol Chem.

[39] Strayer C, Oyama T, Schultz TF, Raman R, Somers D E (2000). Cloning of the *Arabidopsis* clock gene TOC1, an autoregulatory response regulator homolog.. Science.

[40] Onai K, Ishiura M (2005). PHYTOCLOCK 1 encoding a novel GARP protein essential for the *Arabidopsis* circadian clock.. Genes Cells.

[41] Le BH, Cheng C, Bui AQ, Wagmaister JA, Henry KF (2010). Global analysis of gene activity during *Arabidopsis* seed development and identification of seed-specific transcription factors.. Proc Natl Acad Sci USA.

[42] Young TE, Gallie DR (2000). Programmed cell death during endosperm development.. Plant Mol Biol.

[43] Olsen OA (2004). Nuclear endosperm development in cereals and *Arabidopsis* thaliana.. Plant Cell.

[44] Kerk NM, Ceserani T, Tausta SL, Sussex IM, Nelson TM (2003). Laser capture microdissection of cells from plant tissues.. Plant Physiol.

[45] Matsuda F, Miyazawa H, Wakasa K, Miyagawa H (2005). Quantification of indole-3-acetic acid and amino acid conjugates in rice by liquid chromatography-electrospray ionization-tandem mass spectrometry.. Biosci Biotechnol Biochem.

[46] Forestan C, Meda S, Varotto S (2010). ZmPIN1-mediated auxin transport is related to cellular differentiation during maize embryogenesis and endosperm development.. Plant Physiol.

[47] Attia KA, Abdelkhalik AF, Ammar MH, Wei C, Yang J (2009). Antisense phenotypes reveal a functional expression of OsARF1, an auxin response factor, in transgenic rice.. Curr Issues Mol Biol.

[48] Finkelstein R, Reeves W, Ariizumi T, Steber C (2008). Molecular aspects of seed dormancy.. Annu Rev Plant Biol.

[49] Roxrud I, Lid SE, Fletcher JC, Schmidt ED, Opsahl-Sorteberg HG (2007). GASA4, one of the 14-member Arabidopsis GASA family of small polypeptides, regulates flowering and seed development.. Plant Cell Physiol.

[50] Izawa T, Foster R, Nakajima M, Shimamoto K, Chua NH (1994). The rice bZIP transcriptional activator RITA-1 is highly expressed during seed development.. Plant Cell.

[51] Nakase M, Aoki N, Matsuda T, Adachi T (1997). Characterization of a novel rice bZIP protein which binds to the alpha-globulin promoter.. Plant Mol Biol.

[52] Hobo T, Kowyama Y, Hattori T (1999). A bZIP factor, TRAB1, interacts with VP1 and mediates abscisic acid-induced transcription.. Proc Natl Acad Sci U S A.

[53] Kondou Y, Nakazawa M, Kawashima M, Ichikawa T, Yoshizumi T (2008). RETARDED GROWTH OF EMBRYO1, a new basic helix-loop-helix protein, expresses in endosperm to control embryo growth.. Plant Physiol.

[54] Iba K (2002). Acclimative response to temperature stress in higher plants: approaches of gene engineering for temperature tolerance.. Annu Rev Plant Biol.

[55] Hannah MA, Wiese D, Freund S, Fiehn O, Heyer A G (2006). Natural genetic variation of freezing tolerance in Arabidopsis.. Plant Physiol.

[56] Xue H, Chen X, Li G (2007). Involvement of phospholipid signaling in plant growth and hormone effects.. Curr Opin Plant Biol.

[57] Benjamini Y, Yekutieli D (2001). The control of the false discovery rate in multiple testing under dependency.. ANN STAT.

[58] Caldana C, Scheible WR, Mueller-Roeber B, Ruzicic S (2007). A quantitative RT-PCR platform for high-throughput expression profiling of 2500 rice transcription factors.. Plant Methods.

[59] Fu FF, Xue HW (2010). Coexpression analysis identifies Rice Starch Regulator1, a rice AP2/EREBP family transcription factor, as a novel rice starch biosynthesis regulator.. Plant Physiol.

[60] Cline MS, Smoot M, Cerami E, Kuchinsky A, Landys N (2007). Integration of biological networks and gene expression data using Cytoscape.. Nat Protoc.

