# Supplementary figures and images for: Genome-Wide Analysis of the Complex Transcriptional Networks of Rice Developing Seeds
Source: PLoS One. 2012 Feb 17;7(2):e31081. doi: 10.1371/journal.pone.0031081 (PMC3281924; doi:10.1371/journal.pone.0031081)

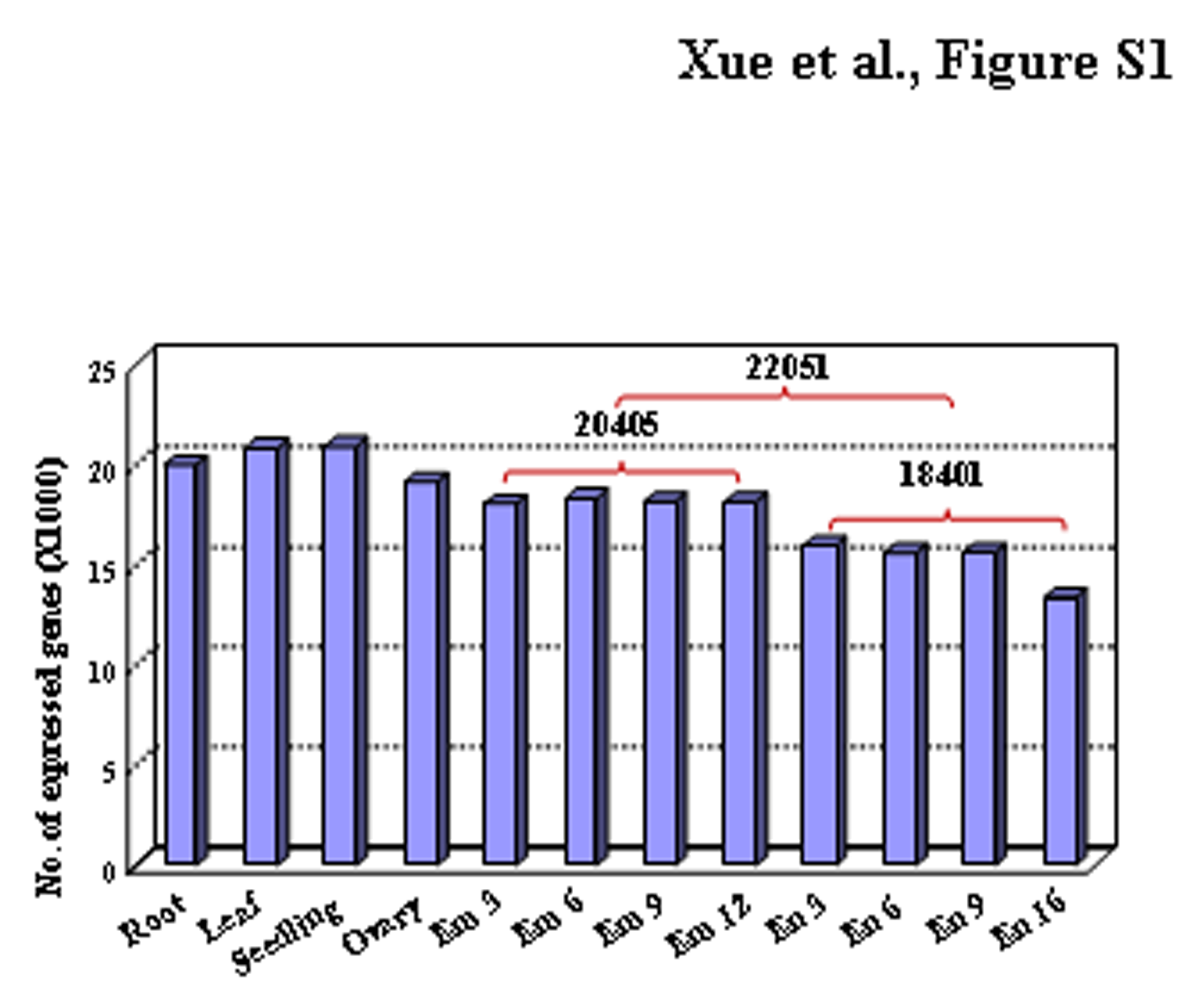

Supplement: Figure S1 — Numbers of genes expressed during seed development. A probe is recorded as expressed when it is eliminated as “P” in two replicates. The numbers indicated the genes expressed in all the stages of embryo (Em) and endosperm (En) development. (TIF) [file pone.0031081.s001.tif]

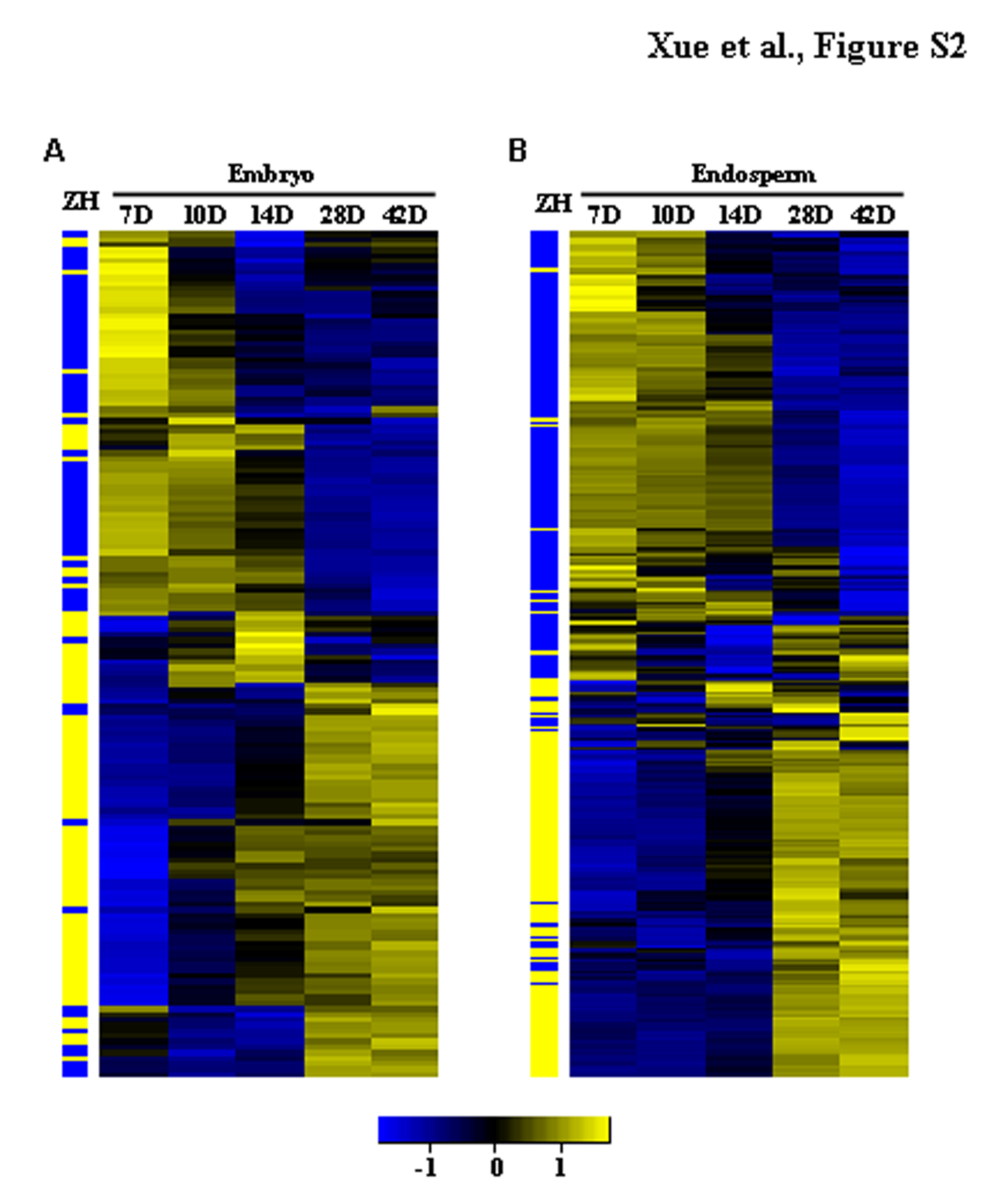

Supplement: Figure S2 — Validation of microarray data. Expression pattern of genes regulated in embryo (A) and endosperm (B) of cultivar Nipponbare are compared with the trends in Zhonghua 11 (ZH). The left vertical bars indicate the individual gene is up-regulated (yellow) or down-regulated (blue) in ZH. “D” indicates days after fertilization (DAF). The data for heatmap were normalized by gene, and the value scale is shown at the bottom. The data of cultivar Nipponbare were from Gene Expression Omnibus (GSE21494). (TIF) [file pone.0031081.s002.tif]

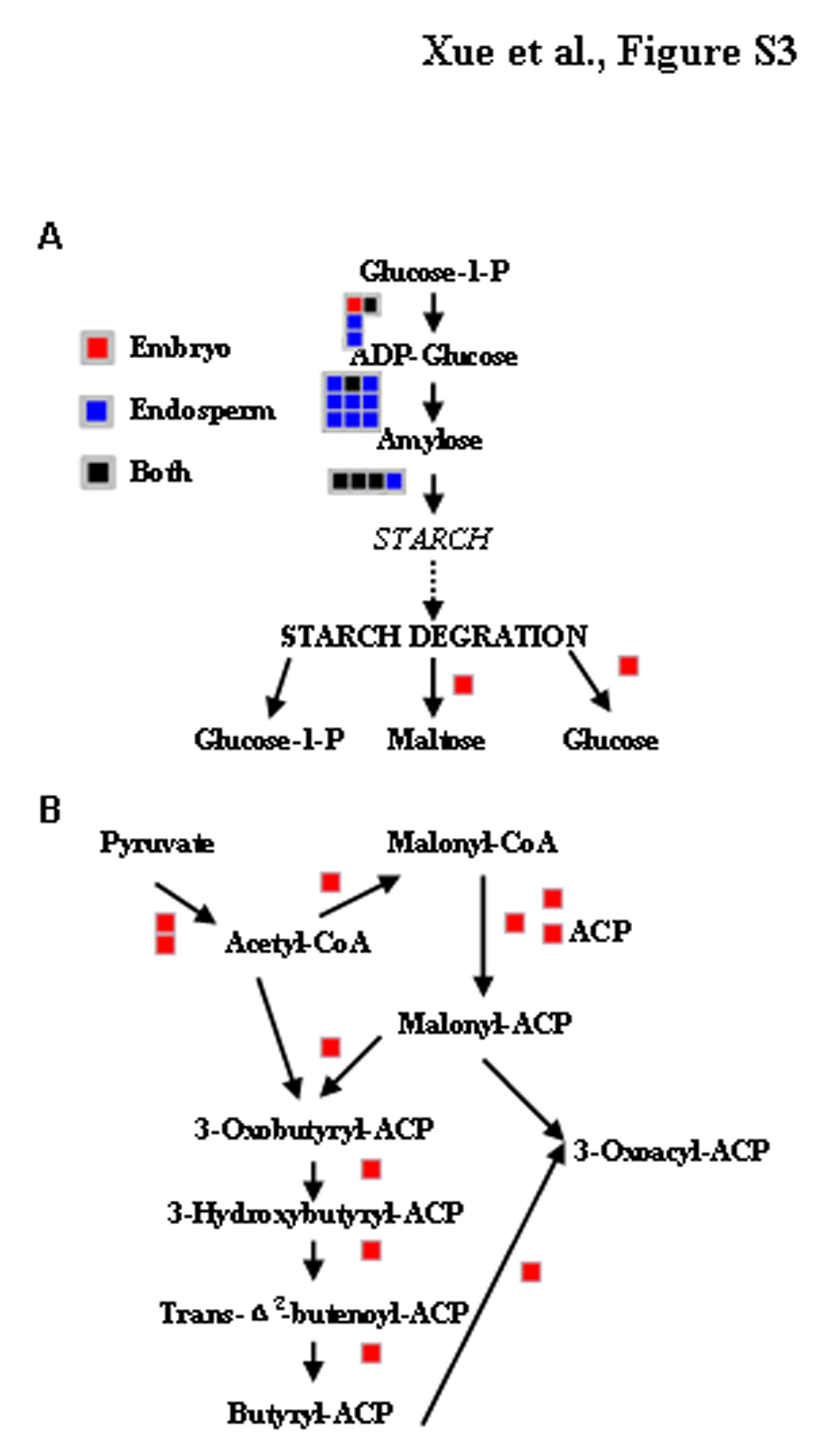

Supplement: Figure S3 — Genes involved in starch (A) and lipid (B) metabolism. Genes are predominantly expressed in embryo (red), endosperm (blue) or both embryo and endosperm (black) are indicated. The annotations of genes are from MAPMAN (http://mapman.gabipd.org). (TIF) [file pone.0031081.s003.tif]

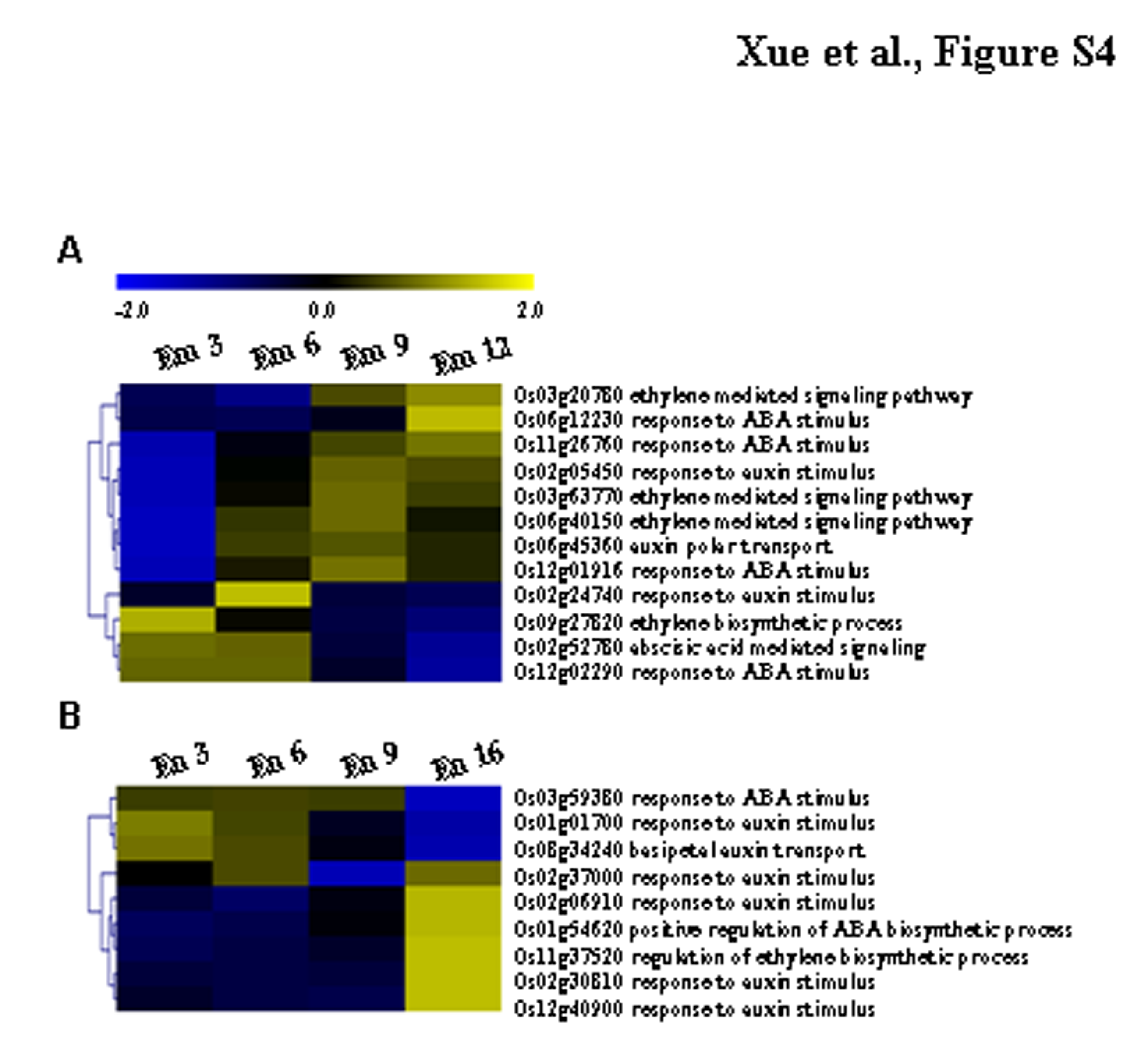

Supplement: Figure S4 — Expression patterns of genes involved in hormone biosynthesis and signaling during embryo (A) or endosperm development (B). The average data of two replicates are calculated, and normalized in Z-score. TIGR MeV was used for visualization. (TIF) [file pone.0031081.s004.tif]
