# Supplementary material for: Genome-Wide Analysis of the Complex Transcriptional Networks of Rice Developing Seeds
Source: PLoS One. 2012 Feb 17;7(2):e31081. doi: 10.1371/journal.pone.0031081 (PMC3281924; doi:10.1371/journal.pone.0031081)
Supplement: Table S1 — Reproducibility of microarray data of each tissue. “PCC” indicates Pearson correlation coefficient of data from the entire array. “ZH” or “HF” indicates the whole seed of Zhonghua 11 or Hanfeng respectively. “C” indicates the low temperature treatment (14°C) for 48 h, “D” indicates days after fertilization (DAF). Refer to Materials and Methods section for details. (DOC) [file pone.0031081.s005.doc]

**Table S1. Reproducibility of microarray data of each tissue.** “PCC” indicates Pearson correlation coefficient of data from the entire array. “ZH” or “HF” indicates the whole seed of Zhonghua 11 or Hanfeng respectively. “C” indicates the low temperature treatment (14^o^C) for 48 h, “D” indicates days after fertilization (DAF). Refer to Materials and Methods section for details.

| Samples | PCC |
| --- | --- |
| Root | 0.960 |
| Leaf | 0.996 |
| Seedling | 0.991 |
| Ovary | 0.970 |
| Embryo 3D | 0.970 |
| Embryo 6D | 0.992 |
| Embryo 9D | 0.983 |
| Embryo 12D | 0.983 |
| Endosperm 3D | 0.970 |
| Endosperm 6D | 0.966 |
| Endosperm 9D | 0.985 |
| Endosperm 16D | 0.959 |
| ZH 4D | 0.879 |
| ZH 6D | 0.929 |
| ZH 4D-2DC | 0.924 |
| ZH 10D | 0.916 |
| ZH 12D | 0.946 |
| ZH 10D-2DC | 0.920 |
| HF 4D | 0.901 |
| HF 4D-2DC | 0.937 |
