# Supplementary material for: Genome-Wide Analysis of the Complex Transcriptional Networks of Rice Developing Seeds
Source: PLoS One. 2012 Feb 17;7(2):e31081. doi: 10.1371/journal.pone.0031081 (PMC3281924; doi:10.1371/journal.pone.0031081)
Supplement: Table S2 — Transcription factors associated with seed development. The TF numbers of each family are shown. “Em” or “En” indicates the genes predominately expressed in embryo or endosperm, respectively. “Both” indicates that genes are predominately expressed in both embryo and endosperm; “Em_T” or “En_T” indicate that genes showing regulated expression pattern during embryo or endosperm development, respectively. “Total Number” indicates the total number of genes in each TF family in genome and “Sum of all TFs” indicates the number of all identified TF genes by hybridization. Chi-square test was performed to test the enrichment of each TF families, if the P value is less than 0.05, the number will be shown in bold. (DOC) [file pone.0031081.s006.doc]

**Table S2. Transcription factors associated with seed development.** The TF numbers of each family are shown. “Em” or “En” indicates the genes predominately expressed in embryo or endosperm, respectively. “Both” indicates that genes are predominately expressed in both embryo and endosperm; “Em_T” or “En_T” indicate that genes showing regulated expression pattern during embryo or endosperm development, respectively. “Total Number” indicates the total number of genes in each TF family in genome and “Sum of all TFs” indicates the number of all identified TF genes by hybridization. Chi-square test was performed to test the enrichment of each TF families, if the P value is less than 0.05, the number will be shown in bold.

| Family | Total number | Em | En | Both | Em_T | En_T | Identified TFs |
| --- | --- | --- | --- | --- | --- | --- | --- |
| bZIP | 92 | 3 | 5 | **4** | **4** | 3 | **19** |
| CCAAT | 46 | 2 | 2 | **8** | 3 | 0 | **15** |
| PHD | 57 | 4 | **5** | 0 | 2 | 1 | **12** |
| AP2-EREBP | 169 | 5 | 3 | 0 | 2 | 2 | 12 |
| bHLH | 151 | 5 | 4 | 1 | 1 | 0 | 11 |
| ABI3VP1 | 55 | **8** | 0 | 2 | 1 | 0 | **11** |
| NAC | 132 | 0 | 3 | **7** | 0 | 0 | 10 |
| C3H | 71 | 2 | 5 | 1 | 1 | 1 | 10 |
| C2H2 | 113 | 4 | 3 | 0 | 0 | 2 | 9 |
| Orphans | 69 | 2 | 2 | 0 | 0 | 3 | 7 |
| HB | 96 | 3 | 3 | 0 | 1 | 0 | 7 |
| MYB | 117 | 3 | 3 | 0 | 1 | 0 | 7 |
| BSD | 10 | 2 | 2 | 0 | 1 | 1 | **6** |
| HSF | 25 | **4** | 0 | 0 | 1 | 1 | **6** |
| GNAT | 37 | 2 | 1 | 0 | 1 | 1 | 5 |
| SET | 32 | 2 | 1 | 0 | 1 | 0 | 4 |
| GRF | 12 | **4** | 0 | 0 | 0 | 0 | **4** |
| TCP | 22 | 0 | 0 | 0 | 1 | **3** | 4 |
| MYB-related | 80 | 0 | 3 | 1 | 0 | 0 | 4 |
| ARF | 25 | 2 | 0 | 1 | 0 | 1 | 4 |
| SNF2 | 37 | 1 | 1 | 0 | 0 | 2 | 4 |
| TRAF | 71 | 0 | 3 | 0 | 0 | 1 | 4 |
| MADS | 69 | 1 | 1 | 0 | 1 | 0 | 3 |
| mTERF | 30 | 1 | 0 | 1 | 0 | 1 | 3 |
| Alfin-like | 10 | 2 | 1 | 0 | 0 | 0 | 3 |
| HMG | 9 | 2 | 0 | 0 | 0 | 1 | **3** |
| FAR1 | 123 | 1 | 2 | 0 | 0 | 0 | 3 |
| SWI/SNF | 11 | 1 | 1 | 1 | 0 | 0 | 3 |
| FHA | 18 | 1 | 2 | 0 | 0 | 0 | 3 |
| WRKY | 105 | 0 | 1 | 0 | 1 | 1 | 3 |
| Jumonji | 14 | 1 | 1 | 0 | 0 | 1 | 3 |
| Other | 438 | 17 | 16 | 5 | 4 | 6 | 48 |
| Sum of all TFs | 2346 | 85 | 74 | 32 | 27 | 32 | 250 |
