# Supplementary material for: Genome-Wide Analysis of the Complex Transcriptional Networks of Rice Developing Seeds
Source: PLoS One. 2012 Feb 17;7(2):e31081. doi: 10.1371/journal.pone.0031081 (PMC3281924; doi:10.1371/journal.pone.0031081)
Supplement: Table S3 — Cis elements associated with seed development. “Ra_G” or “Ra_L” indicate the ratio of cis element in the whole genome or in the gene lists, respectively. “FDR” indicates the FDR corrected P value. (DOC) [file pone.0031081.s007.doc]

**Table S3. Cis elements associated with seed development.** “Ra_G” or “Ra_L” indicate the ratio of cis element in the whole genome or in the gene lists, respectively. “FDR” indicates the FDR corrected P value.

| Cis element | Ra_G | Ra_L | FDR | Key words |
| --- | --- | --- | --- | --- |
| Down-regulated during embryo development | | | | |
| D1GMAUX28 | 0.001 | 0.027 | 0.000 | Auxin, phytochrome |
| GLUTAACAOS | 0.000 | 0.009 | 0.010 | Glutelin, seed, endosperm, |
| Up regulated during embryo development | | | | |
| ABRE2HVA22 | 0.000 | 0.014 | 0.000 | ABRE, G-box |
| ACGTOSGLUB1 | 0.096 | 0.235 | 0.000 | GluB-1, glutelin, endosperm, seed |
| Up-regulated during endosperm development | | | | |
| ACGTOSGLUB1 | 0.096 | 0.213 | 0.000 | GluB-1, glutelin, endosperm, seed |
| ABREMOTIFIIIOSRAB16B | 0.002 | 0.02 | 0.000 | ABA, ABRE |
| ABRERATCAL | 0.597 | 0.75 | 0.000 | ABRE, calcium, |
| SEF1MOTIF | 0.253 | 0.38 | 0.002 | Storage protein, globulin |
| ABREMOTIFAOSOSEM | 0.016 | 0.052 | 0.003 | ABRE, Em, Osem, ABA, VP1, seed |
| ACGTABOX | 0.277 | 0.401 | 0.003 | A-box, G motif, sugar |
| ACGTABREMOTIFAOSOSEM | 0.016 | 0.052 | 0.003 | ABRE, CE3, ABA, VP1, TRAB1 |
| GAGA8HVBKN3 | 0.024 | 0.067 | 0.003 | GAGA, GBP, BBR |
| Predominantly expressed in embryo | | | | |
| ABRE3HVA1 | 0.001 | 0.007 | 0.001 | ABRE, ABA, HVA1, ABRE3, seed, embryo |
| SBOXATRBCS | 0.056 | 0.099 | 0.001 | RbcS, sugar, ABA, ABI4 |
| ANAERO2CONSENSUS | 0.431 | 0.519 | 0.002 | Anaerobic |
| INTRONLOWER | 0.339 | 0.423 | 0.002 | Intron, splice junction |
| CTRMCAMV35S | 0.166 | 0.23 | 0.002 | CaMV 35S, enhancer |
| LECPLEACS2 | 0.234 | 0.303 | 0.004 | Cysteine protease, ethylene, xylanase |
| MYBPLANT | 0.322 | 0.398 | 0.004 | MYB, PAL, phenylpropanoid |
| Predominantly expressed in endosperm | | | | |
| GLUTAACAOS | 0.000 | 0.004 | 0.000 | AACA, glutelin, seed, endosperm |
| GCCCORE | 0.516 | 0.596 | 0.000 | GCC, GCC-box, ERE, JA, Pti4, ERF, PR |
| GLUTEBOX1OSGT2 | 0.000 | 0.000 | 0.000 | Glutelin, Box 1, Gt2, seed, endosperm |
| PYRIMIDINEBOXHVEPB1 | 0.200 | 0.258 | 0.000 | Cysteine proteinase, GA, ABA,aleurone |
| RAV1AAT | 0.860 | 0.907 | 0.000 | RAV1, AP2, VP1, B3, root, leaf, shoot |
| PREATPRODH | 0.389 | 0.453 | 0.000 | Proline, ProDH, bZIP |
| ABRELATERD1 | 0.707 | 0.763 | 0.001 | ABRE, etiolation, erd |
| WRECSAA01 | 0.006 | 0.015 | 0.001 | Wound, AAO, wounding |
| MYB1AT | 0.726 | 0.778 | 0.002 | MYB, rd22BP1, ABA, leaf, seed, stress |
| Highly expressed in both embryo and endosperm | | | | |
| GLUTAACAOS | 0.000 | 0.009 | 0.000 | AACA, glutelin, seed, endosperm |
| AACAOSGLUB1 | 0.000 | 0.002 | 0.000 | GluB-1, glutelin, endosperm, seed |
| GADOWNAT | 0.084 | 0.150 | 0.000 | GA, seed, germination |
| GLUTEBP1OS | 0.000 | 0.002 | 0.000 | Glutelin, seed, endosperm, BP-1, |
| ABREMOTIFAOSOSEM | 0.016 | 0.043 | 0.000 | ABRE, Em, Osem, ABA, VP1, seed |
| ACGTABREMOTIFA2OSEM | 0.229 | 0.317 | 0.000 | ABA, ABRE, motif A, DRE, |
| ACGTABREMOTIFAOSOSEM | 0.016 | 0.043 | 0.000 | ABRE, VP1, TRAB1, Osem, Em, seed |
| ABRELATERD1 | 0.707 | 0.794 | 0.001 | ABRE, etiolation |
