# Supplementary material for: Genome-Wide Analysis of the Complex Transcriptional Networks of Rice Developing Seeds
Source: PLoS One. 2012 Feb 17;7(2):e31081. doi: 10.1371/journal.pone.0031081 (PMC3281924; doi:10.1371/journal.pone.0031081)
Supplement: Table S4 — Identified protein kinases associated with seed development. “P value” is calculated from limma (for predominantly expressed genes) or by ANOVA analysis (for regulated genes). (DOC) [file pone.0031081.s008.doc]

**Table S4. Identified protein kinases associated with seed development.** “P value” is calculated from limma (for predominantly expressed genes) or by ANOVA analysis (for regulated genes).

| Locus | P value | Protein kinase |
| --- | --- | --- |
| Highly in both embryo and endosperm | | |
| Os01g19160 | 7.55E-06 | Receptor-like protein kinase 5 precursor |
| Os03g01850 | 1.52E-07 | Cell division control protein 2 homolog 1 |
| Os03g02680 | 5.68E-06 | Cell division control protein 2 homolog 1 |
| Os03g10940 | 5.46E-06 | Casein kinase II subunit alpha-2 |
| Os05g50120 | 3.58E-06 | Osmpk21-1 |
| Os10g39420 | 3.56E-09 | Calcium-dependent protein kinase, isoform 1 |
| Highly in embryo | | |
| Os01g01410 | 1.93E-05 | Leucine-rich repeat receptor protein kinase EXS precursor |
| Os01g19160 | 2.43E-05 | Receptor-like protein kinase 5 precursor |
| Os01g55450 | 2.13E-07 | CBL-interacting serine/threonine-protein kinase 11 |
| Os01g61590 | 2.69E-07 | Calcium-dependent protein kinase, isoform AK1 |
| Os03g03570 | 1.16E-05 | Leucine-rich repeat transmembrane protein kinase |
| Os03g41460 | 4.36E-06 | Serine/threonine-protein kinase SAPK10 |
| Os03g60710 | 1.73E-05 | Protein kinase APK1B, chloroplast precursor |
| Os04g43710 | 2.95E-06 | Calcium-dependent protein kinase |
| Os07g01710 | 8.39E-09 | Phytosulfokine receptor precursor |
| Os09g15700 | 2.73E-05 | Receptor-like protein kinase 5 precursor |
| Os10g06740 | 1.05E-06 | Receptor-like protein kinase precursor |
| Os10g19160 | 2.04E-05 | Receptor kinase |
| Os12g44090 | 5.01E-05 | Leucine-rich repeat transmembrane protein kinase |
| Regulated in embryo | | |
| Os01g20900 | 9.69E-04 | OsWAK4 - osWAK receptor-like cytoplasmic kinase (osWAK-RLCK) |
| Os01g26160 | 9.86E-04 | Hst |
| Os01g38950 | 1.32E-04 | Casein kinase I isoform delta-like |
| Os03g02680 | 6.90E-04 | Cell division control protein 2 homolog 1 |
| Os07g39520 | 6.52E-05 | Serine/threonine-protein kinase WNK2 |
| Os12g06490 | 7.01E-04 | Serine/threonine-protein kinase WNK3 |
| Highly in endosperm | | |
| Os01g19160 | 6.76E-07 | Receptor-like protein kinase 5 precursor |
| Os01g49529 | 1.13E-06 | OsWAK10a - osWAK receptor-like cytoplasmic kinase (osWAK-RLCK) |
| Os01g60910 | 2.38E-08 | CBL-interacting serine/threonine-protein kinase 15 |
| Os02g34430 | 6.54E-05 | BRI 1-associated receptor kinase 1 |
| Os03g17550 | 2.09E-05 | Serine/threonine-protein kinase NAK |
| Os03g17980 | 1.99E-06 | Carbon catabolite derepressing protein kinase |
| Os03g35600 | 6.22E-08 | Serine/threonine-protein kinase receptor precursor |
| Os03g43590 | 2.11E-05 | LSTK-1-like kinase |
| Os03g50390 | 1.82E-05 | AGC_AGC_other_gwld.2 |
| Os04g46320 | 6.26E-05 | Protein Kinase-like protein TMKL1 precursor |
| Os05g06990 | 6.89E-07 | TKL_IRAK_crrlk1l-1.10 |
| Os06g07070 | 1.02E-07 | BRI 1-associated receptor kinase 1 |
| Os06g08280 | 8.38E-05 | Protein kinase domain containing protein |
| Os08g06060 | 5.35E-10 | Mitogen-activated protein kinase homolog 4 |
| Os08g35220 | 6.44E-05 | Cell division cycle 2-related protein kinase 7 |
| Os08g37800 | 1.40E-06 | Carbon catabolite derepressing protein kinase |
| Os10g29540 | 4.58E-05 | EDR1 |
| Regulated in endosperm | | |
| Os01g07560 | 6.65E-04 | Receptor-like protein kinase precursor |
| Os02g56560 | 2.55E-04 | Casein kinase I isoform delta-like |
| Os03g41460 | 9.28E-04 | Serine/threonine-protein kinase SAPK10 |
| Os05g34270 | 9.89E-05 | BRI1-associated receptor kinase 1 |
| Os08g34240 | 1.52E-04 | CBL-interacting serine/threonine-protein kinase 15 |
| Os12g02250 | 3.95E-04 | Mitogen-activated protein kinase |
| Os12g44090 | 3.83E-04 | Leucine-rich repeat family protein |
