# Supplementary material for: Genome-Wide Analysis of the Complex Transcriptional Networks of Rice Developing Seeds
Source: PLoS One. 2012 Feb 17;7(2):e31081. doi: 10.1371/journal.pone.0031081 (PMC3281924; doi:10.1371/journal.pone.0031081)
Supplement: Table S5 — Transcription factors (TFs) those are down-regulated in Zhonghua 11 seeds by low temperature at early stage. “Type” indicates the expression pattern of TF genes during seed development, including En (endosperm), Em (embryo), Both (endosperm and embryo) and En_T (regulated expression pattern during endosperm development in time course). (DOC) [file pone.0031081.s009.doc]

**Table S5. Transcription factors (TFs) those are down-regulated in Zhonghua 11 seeds by low temperature at early stage.** “Type” indicates the expression pattern of TF genes during seed development, including En (endosperm), Em (embryo), Both (endosperm and embryo) and En_T (regulated expression pattern during endosperm development in time course).

| Gene | Ratio | Family | Type | Homolog genes  in Arabidopsis |
| --- | --- | --- | --- | --- |
| Os03g08460 | 51.971 | AP2-EREBP |  | RAP2.12 |
| Os06g39240 | 7.035 | MBF1 | En | MBF1C |
| Os02g58210 | 6.078 | Jumonji | Em | jmjC |
| Os09g28440 | 4.331 | AP2-EREBP |  | RRTF1 |
| Os01g74020 | 3.912 | G2-like |  | PCL1 |
| Os06g15480 | 3.542 | bZIP | En_T | PAN |
| Os08g31930 | 3.45 | PHD |  | Zinc finger |
| Os01g74590 | 3.247 | MYB | En | ATMYBR1 |
| Os09g35790 | 3.211 | HSF |  | AT-HSFB2B |
| Os08g09690 | 3.151 | CCAAT-HAP2 |  | NF-YA4 |
| Os02g36924 | 2.967 | MADS |  | AGL16 |
| Os02g40510 | 2.571 | Pseudo ARR-B |  | TOC1 |
| Os01g04800 | 2.448 | AP2-EREBP |  | RAV2 |
| Os05g34310 | 2.082 | NAC | Both | anac025 |
