# Supplementary material for: Genome-Wide Analysis of the Complex Transcriptional Networks of Rice Developing Seeds
Source: PLoS One. 2012 Feb 17;7(2):e31081. doi: 10.1371/journal.pone.0031081 (PMC3281924; doi:10.1371/journal.pone.0031081)
Supplement: Table S6 — Involved biological processes of genes with differential expression in Hanfeng variety. aGenes were highly expressed in Zhanghua 11 (ZH), bgenes were highly expressed in Hanfeng (HF). Chi-square test was performed to test the enrichment of each GO term. The data indicates −log10 (P value). “-” indicates that the genes are not enriched in the GO term. (DOC) [file pone.0031081.s010.doc]

**Table S6. Involved biological processes of genes with differential expression in Hanfeng variety.** ^a^Genes were highly expressed in Zhanghua 11 (ZH), ^b^genes were highly expressed in Hanfeng (HF). Chi-square test was performed to test the enrichment of each GO term. The data indicates -log10 (P value). “-” indicates that the genes are not enriched in the GO term.

| GO ID | GO term | Pre treatment | | | Treated | |
| --- | --- | --- | --- | --- | --- | --- |
|  |  | ZH^a^ | | HF^b^ | ZH^a^ | HF^b^ |
| GO:0019915 | Sequestering of lipid | - | | 6.717 | - | 4.422 |
| GO:0006635 | Fatty acid β-oxidation | - | | 5.451 | - | 3.583 |
| GO:0006096 | Glycolysis | - | | 3.056 | - | 1.984 |
| GO:0019575 | Sucrose catabolic process | - | | - | - | 7.267 |
| GO:0009073 | Aromatic amino acid family biosynthetic process | - | | - | - | 6.087 |
| GO:0005986 | Sucrose biosynthetic process | - | | - | - | 6.087 |
| GO:0008654 | Phospholipid biosynthetic process | - | | - | - | 5.495 |
| GO:0006800 | Oxygen and ROS metabolic process | - | | - | 5.951 | 4.102 |
| GO:0046488 | Phosphatidylinositol metabolic process | 11.102 | | - | - | - |
| GO:0044267 | Cellular protein metabolic process | 6.120 | | - | - | - |
| GO:0016310 | Phosphorylation | 5.882 | | - | - | - |
| GO:0006139 | Nucleobase, nucleic acid metabolism | 3.465 | | - | 3.782 | - |
| GO:0006075 | 1,3-beta-glucan biosynthetic process | - | | - | 10.532 | - |
| GO:0009311 | Oligosaccharide metabolic process | - | | - | 3.079 | - |
|  |  |  | |  |  |  |
| GO:0007049 | Cell cycle | 5.092 | | - | 5.552 | - |
| GO:0000910 | Cytokinesis | 5.882 | | - | - | - |
| GO:0000226 | Microtubule cytoskeleton organization and biogenesis | | - | - | 9.892 | - |
| GO:0007018 | Microtubule-based movement | 2.986 | | - | - | - |
| GO:0006298 | Mismatch repair | 8.554 | | - | 9.326 | - |
| GO:0006913 | Nucleocytoplasmic transport | 8.554 | | - | 9.326 | - |
| GO:0006904 | Vesicle docking during exocytosis | - | | - | 3.079 | - |
| GO:0045454 | Cell redox homeostasis | - | | - | 2.865 | 1.949 |
| GO:0009992 | Cellular water homeostasis | - | | - | 3.137 | - |
| GO:0042542 | Response to hydrogen peroxide | - | | - | 13.225 | 2.422 |
| GO:0010020 | Chloroplast fission | - | | 14.922 | - | 9.821 |
|  |  |  | |  |  |  |
| GO:0035194 | Posttranscriptional gene silencing by RNA | 10.330 | | - | - | - |
| GO:0030422 | RNA interference, production of siRNA | 10.330 | | - | - | - |
| GO:0006396 | RNA processing | - | | 2.195 | - | 5.216 |
| GO:0006364 | RNA processing | 5.458 | | - | - | - |
|  |  |  | |  |  |  |
| GO:0000059 | Protein import into nucleus, docking | 8.554 | | - | - | - |
| GO:0006512 | Ubiquitin cycle | - | | 4.252 | - | - |
| GO:0006486 | Protein amino acid glycosylation | - | | 3.003 | - | 1.949 |
| GO:0006470 | Protein amino acid dephosphorylation | - | | - | 2.722 | - |
| GO:0009306 | Protein secretion | - | | - | - | 5.240 |
| GO:0006511 | Ubiquitin-dependent protein catabolic process | - | | - | 1.633 | 2.334 |
