# Supplementary material for: Genome-Wide Analysis of the Complex Transcriptional Networks of Rice Developing Seeds
Source: PLoS One. 2012 Feb 17;7(2):e31081. doi: 10.1371/journal.pone.0031081 (PMC3281924; doi:10.1371/journal.pone.0031081)
Supplement: Table S7 — Primers used in real time quantitative PCR (qRT-PCR) analysis. “S” and “A” indicate the sense or antisense primer respectively. (DOC) [file pone.0031081.s011.doc]

**Table S7. Primers used in real time quantitative PCR (qRT-PCR) analysis.** “S” and “A” indicate the sense or antisense primer respectively.

| Gene | Primer | Sequences(5’-3’) |
| --- | --- | --- |
| Os10g11580 | S | CTGAAGGAAGTGATGGTGCCG |
|  | A | CTTGTGCTGTTTCGCCTTTCG |
| Os02g03030 | S | GGCGGACAGGTTCATCAAGG |
|  | A | AAGCGGACGCACTTGAGATT |
| Os06g15480 | S | AGCACCAGCGGCACATCAAC |
|  | A | GGCGGAACACCTGGTCGTAG |
| Os01g53220 | S | GGGCCAGATGATGGCGTTCC |
|  | A | CAGGGATCGGACCCGTTGTT |
| Os02g49370 | S | TCGGTGGGATGCTCAAGTCC |
|  | A | GGCGGCATGAGCTGGTGGAA |
| Os08g09230 | S | ATACTGCGACAAAGCCACAACT |
|  | A | CAGCATTCTTGCCTTCAACCAC |
| Os12g27830 | S | GGCAAGGGAAGCGTTGTCTCA |
|  | A | CCACCAGCCTCAGCCATCTTC |
| Os02g16820 | S | TTAATGCCCATAGCCTGGTGTA |
|  | A | AAGCATTAGCGATCACATCCACT |
| Os05g36290 | S | ATCCTTGTATGCTAGCGGTCGA |
| (ACTIN1) | A | ATCCAACCGGAGGATAGCATG |
